# Paternal exposure to a common herbicide alters the behavior and serotonergic system of zebrafish offspring

**DOI:** 10.1371/journal.pone.0228357

**Published:** 2020-04-10

**Authors:** Simon D. Lamb, Jolyn H. Z. Chia, Sheri L. Johnson

**Affiliations:** Department of Zoology, University of Otago, Dunedin, Otago, New Zealand; University of Vienna, AUSTRIA

## Abstract

Increasingly, studies are revealing that endocrine disrupting chemicals (EDCs) can alter animal behavior. Early life exposure to EDCs may permanently alter phenotypes through to adulthood. In addition, the effects of EDCs may not be isolated to a single generation − offspring may indirectly be impacted, via non-genetic processes. Here, we analyzed the effects of paternal atrazine exposure on behavioral traits (distance moved, exploration, bottom-dwelling time, latency to enter the top zone, and interaction with a mirror) and whole-brain mRNA of genes involved in the serotonergic system regulation (*slc6a4a*, *slc6a4b*, *htr1Aa*, *htr1B*, *htr2B*) of zebrafish (*Danio rerio*). F0 male zebraFIsh were exposed to atrazine at 0.3, 3 or 30 part per billion (ppb) during early juvenile development, the behavior of F1 progeny was tested at adulthood, and the effect of 0.3 ppb atrazine treatment on mRNA transcription was quantified. Paternal exposure to atrazine significantly reduced interactions with a mirror (a proxy for aggression) and altered the latency to enter the top zone of a tank in unexposed F1 offspring. Bottom-dwelling time (a proxy for anxiety) also appeared to be somewhat affected, and activity (distance moved) was reduced in the context of aggression. *slc6a4a* and *htr1Aa* mRNA transcript levels were found to correlate positively with anxiety levels in controls, but we found that this relationship was disrupted in the 0.3 ppb atrazine treatment group. Overall, paternal atrazine exposure resulted in alterations across a variety of behavioral traits and showed signs of serotonergic system dysregulation, demonstrating intergenerational effects. Further research is needed to explore transgenerational effects on behavior and possible mechanisms underpinning behavioral effects.

## Introduction

Exposure to endocrine and neuroendocrine disrupting chemicals (EDCs) are reported to induce a variety of aberrant behaviors (see reviews: [1,2]), particularly when individuals are exposed early in life [3–8]. EDCs interfere with the molecular mechanisms that underpin behavior, such as gene expression, hormone levels, neurotransmitter levels, and the molecular machinery that mediates between these inputs [9–15]. The effects of EDC exposures are predominantly implicated in the disruption of typical reproductive behaviors (i.e. courtship and parental behavior; [2,3,16]), but non-reproductive behaviors, such as anxiety, aggression and risk-taking behavior also appear to be strongly affected [4,17–22,22–25]).

Increasingly, studies are revealing that the effects of EDCs may not be isolated to a single generation–offspring may indirectly be impacted through their parents via non-genetic processes (i.e. the transmission of parental phenotypic or environmental variation to offspring that do not stem from changes in DNA sequence; [4,6,25–29]). For example, female F3 descendants of mice embryonically exposed to the EDC, vinclozolin, prefer males without a history of exposure [27], and parental exposure to bisphenol A in medaka (*Oryzias latipes*) result in larval offspring with reduced locomotory behavior [30]. Maternal contribution to phenotypic variation via non-genetic inheritance is well documented (e.g. the transfer of maternal glucocorticoids to developing eggs [31–34] or maternal care; [35–40]). For instance, female sticklebacks (*Gasterosteus aculeatus*) exposed to predation risk produced eggs that were larger and had elevated cortisol levels, moreover, their juvenile offspring exhibited tighter shoaling behavior [33]. However, the understanding of the paternal contribution is more limited. Recent work is recognizing that sperm can be an important source contributing to non-genetic inheritance [41,42]. For example, offspring sired by zebrafish males experiencing increased sperm competition exhibit faster hatching rates, but reduced survival [43], and learned fear responses can be inherited via sperm in mice [41]. Furthermore, Anway et al. [44] found that transgenerational exposure of vinclozolin (through the male-line), results in increased incidents of infertility (up to four generations) in rats.

Non-genetic mechanisms are also hypothesized to contribute to animal behavior differences [45–47]. Thus, the behavioral phenotype of unexposed descendants, in combination with EDC exposure, may be detrimentally impacted. For instance, parental or transgenerational exposure to 17α-ethinylestradiol in zebrafish (*Danio rerio*) and guppies (*Poecilia reticulata*) results in heightened anxiety phenotypes in zebrafish F1 offspring and in F1 and F2 generations in guppies [4,25].

Here, we investigate whether exposure of zebrafish males to the herbicide, atrazine, during early juvenile development affects the behavioral traits of their unexposed F1 progeny at adulthood. Atrazine (2-chloro-4-ethylamino-6-isopropylamino-1,3,5-triazine) is a commonly used herbicide to control weeds in a variety of crops [48,49], but often leeches into the aquatic environment [35]. The maximum allowable atrazine concentrations in drinking water range from 0.1 to 5 ppb in most regions, particularly in Asia and the USA, though environmental concentrations of 30 ppb in ground and surface waters have been reported [50], and atrazine can sometimes be found in much higher concentrations, even over 300 ppb [51].

In addition, atrazine has been found to possess neuroendocrine disrupting properties [5,8,52–55]. The mechanism underlying atrazine neuroendocrine disruption still remains to be resolved, but is implicated in disrupting a variety of pathways including the hypothalamic-pituitary gonadal axis [56–59], monoaminergic systems within the central nervous system [5,54,60–62], alterations to the cyclic adenosine monophosphate (cAMP) dependent signaling pathway [63–67], as well as many epigenetic mechanisms, including microRNAs and expression of DNA methyltransferases (see [55] for more details; [7,68]). Previous research has demonstrated that atrazine exposure in fish can reduce mating behavior (male-male aggression in a courtship context; [3,68]) and alter some aspects of sociability [69], personality phenotypes in crayfish [70], increased anxiety and reduced spatial memory and learning in rodents [54,67,71], but to our knowledge, no study has yet explored how atrazine may affect behavioral traits of unexposed offspring. We hypothesized that if changes in progeny behavior (relative to controls) occurred, this would provide evidence that the herbicide atrazine has the capacity to influence behavior across generations, and these changes are likely to be attributed to a non-genetic, paternal component of inheritance. Based on literature on zebrafish and other species (i.e. mouse and guppy), we predicted that exposure to increasing levels of atrazine increases bottom-dwelling time (a proxy for anxiety) and activity levels [5,60,72] and decreases latency to enter the top zone of a tank (a proxy for boldness), interaction with a mirror (a proxy for aggression) and exploration [3,68].

In addition to behavioral outcomes, we also assess whether behavioral changes are underpinned by changes in the mRNA transcript of genes involved in regulating the serotonergic system, as recent transcriptomic studies from adult zebrafish embryonically exposed to atrazine have highlighted the serotonergic system as a potential target of developmental atrazine exposure [5,73]. As candidate genes, this study selected three serotonin (5-HT) receptor genes *htr1Aa*, *htr1B*, and *htr2B*, as well as the two 5-HT transporter genes *slc6a4a* and *slc6a4b* for their involvement in the regulation of the serotonergic system and in anxiety related behaviors [74–76]. Thus, for the second part of this experiment we specifically focus on bottom-dwelling time (anxiety) behavior. The three 5-HT receptors function by propagating neurotransmission via the relaying of 5-HT activity and are involved in controlling 5-HT release [77,78]. The two 5-HT transporters [79] function by regulating the strength and duration of neurotransmission [77]. Drugs that target the functional proteins and expression levels of these genes are implicated in anxiety-like behaviors [76]. Moreover, single nucleotide polymorphism of 5-HT transporters has been associated with behavioral differences in other taxa (e.g. [80–83]).

## Material and methods

### Experimental overview

Juvenile F0 zebrafish were exposed to atrazine at environmentally relevant concentrations of 0.3, 3 or 30 part per billion (ppb; [5,6,35]) for 10 days during sexual differentiation (27–37 days post fertilization; dpf). F1 offspring were produced from atrazine exposed and control males, via *in vitro* fertilization (IVF: modified from Johnson et al. [84]) with unexposed females, creating full-sibling families. F1 behavior (see below) was assayed at adulthood (behavior was also tested on the adult F0 fish [85]). Whole-brains of control and 0.3ppb fish were used to determine mRNA transcript number of candidate genes involved in the regulation of the serotonergic system (*slc6a4a*, *slc6a4b*, *htr1Aa*, *htr1B*, *htr2B)*. The 0.3ppb progeny were selected as we had previously found that bottom-dwelling time (anxiety) was significantly increased in the F0 0.3ppb treatment [85] and because 0.3 ppb represents a typical environmental concentration of atrazine exposure [35]. All experiments and handling of the animals were performed according to the New Zealand Animal Welfare Act and approved by the University of Otago Animal Ethics Committee.

### Breeding and husbandry

Breeding, husbandry and atrazine exposure took place within the Otago Zebrafish Facility (OZF), a temperature-controlled facility with a 14h (0800–2200 h) dawn-dusk light cycle. All fish were housed in 3.5 L tanks (except during atrazine exposure) on a Tecniplast ZebTECH zebrafish housing system (Tecniplast). The conductivity, temperature and pH were maintained between 390–458 μS, 25.2–26.1°C and 7–7.8 pH, respectively. Zebrafish were fed *ad libitum*, twice daily with dry food (ZM000-400, size-dependent) and once daily with live rotifer (*Brachionus* spp; 4–10 dpf) or live *Artemia* (*Artemia salina;* 10dpf and beyond).

F0 embryos were produced by group spawning wildtype AB zebrafish (24 females, aged ~10 months post fertilization; mpf and 34 males aged ~11 mpf) using a Tecniplast iSpawn Breeding System (Tecniplast) and exposed to atrazine at 27–37 days post fertilization (see below). Unexposed F1 progeny were then produced when F0 fish reached sexual maturity, ~3–4 months post fertilization (106–141 dpf), using IVF (modified from Johnson et al. [84]) with untreated AB females (aged ~8 mpf). Three F0 males per treatment were randomly selected producing three full-sibling F1 families per treatment; 12 families in total). Both F0 and F1 embryos were incubated in petri dishes (90 mm diameter) with E3 media (Cold-Spring-Harbor-Protocols) at 28.4°C until 4dpf. After four days, hatched fry were moved into 3.5 L tanks with AquaOne, 5 parts per trillion Aquaria Salt solution (synthetic sea salt).

### Atrazine exposure

A stock solution of atrazine (Sigma-Aldrich; CAS 1912-24-9; 5 mg in 200 mL) was prepared three days prior to exposures. At 27 dpf, F0 zebrafish fry (80 fry per treatment) were exposed to atrazine (at 0ppb 0.3ppb, 3ppb or 30ppb) in 500 mL of system water using 600 mL (80 mm diameter) glass beakers. The atrazine exposure regime lasted 10 days (26/02/17 through to the 8/03/17). This window corresponds with the first ~10 days of sexual differentiation in zebrafish in our OZF facility [86,87]. Fry were fed once a day during the exposure regime and water was changed daily (100% water change; from day 2 until day 10); thus, concentrations of atrazine were also renewed daily–the larvae were sieved, rinsed and placed in new beakers of the appropriate atrazine concentration. At the end of 10 days, fry were moved back onto the OZF system. While we did not analytically verify atrazine concentrations, the exposures were conducted in a closed system and this same exposure regime has been used in several studies [3,5,6,68] where the concentrations were verified. Furthermore, concentrations used by Shenoy [3,68] (1 ppb and 15 ppb) were found to decrease negligibly over 4–7 days without renewal in a closed system (to 0.26 ppb and 12.98–13.45 ppb, respectively). However, we recognize that there could be some error in using nominal (non-measured concentrations), though the concentrations will still vary by orders of magnitude.

### F1 behavioral assays

F1 fish were exposed to a series of behavioral assays: a novel arena, a novel object and a mirror test, all in the same tanks [88–91]. Behavioral measurements were recorded during each test (see below for details about behavioral measures recorded and background of behavioral assays used) by live-tracking fish using EthoVision XT behavioral tracking software version 11.5 [92].

The novel arena, the novel object and the mirror tests were run consecutively in the same tank with each assay lasting 10 min (600 s) each, with three fish assayed simultaneously in three separate tanks (length = 30 cm, width = 15.5 cm, height = 27 cm filled with 8 L of system water to a depth of 19.5 cm; Fig A in S1 File). A flat mirror (vertical length = 19 cm, width = 15 cm) was fixed to the outside wall of each tank, which was covered with a removable, hard plastic opaque barrier during the novel arena and novel object tests. White film was fixed to the bottom and side of each tank wall to limit light and reflection during filming (except on the side where the mirror was placed). The back of the tank was fixed with white and opaque plastic. The novel object was an orange rubber bung (measuring 3.2 cm long, 4.3 cm wide at the bottom and 3.7 cm wide at the top) attached to plastic fishing wire hanging in the middle of the arena (at an approximate depth of 6.9 cm below the surface of the water). Tanks were lit 30cm above the tanks with one 240 V 48LED aluminum light strip and from behind using three Godox LED170 lights (31–35.5 cm away) to provide diffusing light to increase fish contrast during filming. Fish were filmed with a Basler acA1300-60/gc GigE camera with a 4.4–11 mm lens placed about 112 cm away from the row of tanks and live-tracked using EthoVision XT [92]. Live-tracking with EthoVision XT started 10–30 s into each trial (e.g., after the fish were placed in the novel arena, 10–30 s after the novel object was added and 10–30 s after the opaque barrier covering the mirror was removed).

In total, 190 F1 fish were assayed (control males = 21, females = 28; 0.3ppb males = 22, females = 24; 3ppb males = 18, females = 30; 30ppb males = 18, females = 29), however, there were eight instances during the novel object assay where tracking was unable to be accurately established due to experimental error. These data (three controls (two males, one female), one 0.3ppb male, three 3ppb fish (one male and two females) and one 30ppb female) were therefore excluded from subsequent analyses on behavioral measures recorded during the novel object test.

### Background information of assays and behavioral measures recorded

The novel arena test is routinely used to measure anxiety, activity, and exploration (predominately after chemical exposures in fish; [88,93]). Zebrafish, when exposed to an unfamiliar environment (i.e. the novel arena), tend to dive to the bottom of the test arena and tend to remain in the lowest portion (bottom-dwelling behavior), avoiding the higher portions of the water column, and then the fish usually begin to explore the other areas of the tank after a few minutes [88,89]. Thus, we measured anxiety in the novel arena by the amount of total time spent (s) in the bottom zone (i.e. the total time spent in the lowest four zones superimposed within EthoVision XT; Fig A in S1 File). Exploration was measured by the standard deviation of time spent (s) in each of 12 zones (Fig A in S1 File) during the novel arena test (i.e. an explorative fish would have a standard deviation closer to zero, meaning a fish spent an equal amount of time in all areas of the tank, a high standard deviation indicates the fish was less explorative, preferring to spend the majority of the assay in a few areas of the tank [91]). We measured the latency (s) to enter the top zone (taken as the first moment that a fish entered the top portion of the tank; i.e. the first time a fish entered into one of the four highest zones superimposed within EthoVision XT).

The novel object test was considered a complementary assay to the novel arena test. When exposed to a novel object, zebrafish show a tendency to avoid the foreign object, exhibit bottom-dwelling behavior, and after a certain amount of time, the zebrafish should begin to inspect the object [91,94,95]. We measured the amount of time spent in the bottom zone (s) throughout the assay and novel object approach (1), approached the novel object (entered the zone surrounding the novel object), or (0), did not approach the novel object. (Fig A in S1 File). A zebrafish approaching the novel object (orange rubber stopper) within 1–1.5 body lengths is considered a measure of boldness and is interpreted as a predator inspection behavior [89,91,94–96].

Finally, the mirror test is typically used in assessing an individual’s level of aggression (e.g. [90,97,98]. Zebrafish are unable to recognize their reflection and are thought to perceive their mirror image as an intruder [90,99]. The time spent (s) interacting with the mirror (within the mirror zone; Fig A in S1 File) was recorded. Across all three assays, activity was measured by the total distance moved (cm).

Previous work in our lab has shown that the repeatability (the intra-class correlation values, i.e. animal personality) of zebrafish behavior (for the behavioral measures described above) is dependent on an initial exposure to the assay regime, i.e., repeatability was low between the initial test and additional tests, but repeatability was high from the second assay onwards [91]. Considering these findings, an ‘assay experience’ was provided (i.e. fish were exposed to the assay regime twice, but the data presented is that of the second assay trials). The assay regime proper was conducted 7 days after the assay experience. The repeatability of behavior exhibited during the mirror tests (i.e. time spent interacting with the mirror) does not appear to be dependent on an initial exposure [85], but it was included to maintain consistency with the other behavioral tests. Because fish were not individually marked, we were unable to establish changes in behavior of individuals between trials.

### F1 brain collection

Immediately after phenotyping (the second trials i.e. the assay regime proper) whole-brain tissue was collected from the two males and two females (per each family of the control and 0.3ppb treatment) that spent the most and the least amount of time at the bottom zone during the novel arena assay (a high and low anxiety phenotype). We selected the most and least anxious fish to capture a wide range of behavioral expression and potentially any underlying signal in mRNA gene expression. Once selected, fish were then euthanized and dissected in 1X PBS (total number of fish dissected = 24 fish per treatment). There were two instances in the control group (two males) where we were unable to extract brain tissue. Once extracted, whole-brain tissue was immediately stored in 50μL of RNAlater (Invitrogen) and subsequently stored at −30°C. The 0.3ppb treatment was chosen for comparison against controls as it represents a typical environmentally relevant level of atrazine exposure [35] and because we previously found that F0 fish exposed to the 0.3ppb atrazine treatment exhibited significant increases in bottom-dwelling [85].

### Quantitative real-time PCR (qPCR)

RNA was extracted from whole-brain tissue using a modified protocol from the Norgen Biotek RNA kit and using TRIzol reagent (Ambion) for initial homogenization. Extracted RNA was purified using Turbo DNase (Invitrogen). The RNA was reverse transcribed to obtain cDNA using 400 ng of RNA and a High Capacity cDNA Reverse Transcription kit (Applied Biosystems). The parameters for reverse transcription (following the manufacture’s protocol) consisted of one cycle of 25°C for 10 min, 37°C for 120 min and 85°C for 5 min (ThermoFisher).

Whole-brain mRNA expression of genes encoding for *slc6a4a*, *slc6a4b*, *htr1Aa*, *htr1B*, and *htr2B* (Table A in S1 File) was conducted using QuantStudio 5 (Thermofisher). The total reaction volume was 10 *μ*L, containing 5 *μ*L SYBR green (Takara), 0.5 *μ*L (10 *μ*M) forward primer, 0.5 *μ*L (10 *μ*M) reverse primer, 2.8 *μ*L MilliQ water, 0.2 *μ*L ROX and 1 *μ*L (10 ng/*μ*L) of cDNA. For each primer, a gradient temperature assessment was carried out from 60–64°C in order to find the optimal annealing temperature. Each cycle of qPCR was 95°C for 2 min, 95°C for 5 s, optimal annealing temperature of gene primer for 10 s, 72°C for 5 s, 95°C for 1 min, 55°C for 30 s, 95°C for 30 s, for 40 cycles per plate. qPCR standards for each gene were made using pooled cDNA (of all 46 samples; 22 controls and 24 0.3ppb treatment offspring) and serially diluted (6X) at a ratio of 1:3 dilution. Efficiency of standards for all qPCR runs was between 95–105%. *b-actin* was validated to be a suitable reference gene.

### Statistical analysis

All statistical analyses were conducted in R, version 3.5.0 [100]. All analyses on behavior described below contained the two main predictors, treatment (i.e. controls, 0.3ppb, 3ppb, 30ppb) and sex (male and female) and an interaction term between treatment and sex. The interaction term was removed if non-significant and the data were re-analyzed without it. All behavioral measures were analyzed individually with linear mixed effects models (LMMs: the time spent in the bottom zone of the novel arena/ novel object test, exploration in the novel arena, time spent interacting with the mirror and the total distance moved in each assay) or with generalized linear mixed effects models (GLMMs: the latency to enter the top zone of the novel arena using a gamma error structure (log-link function), and approaching or not approaching the novel object using a binomial error structure (logit-link function)), using the package ‘*lme4*’ version 1.1–1.3 [101]. The assumptions of a GLMM with a gamma error structure are that zeros are unable to be analyzed (therefore if a fish was in the top zone at the start of the trial; it was given a latency of 0.1 s, if the fish never entered the top zone; it was given a latency of 600 s (the total duration of the assay). In all models, family identity was included as a random effect to account for genetic influences.

To test if an bottom-dwelling time (a proxy for anxiety) was underpinned by mRNA transcript number and whether or not paternal atrazine exposure altered the relationship of mRNA transcripts between treatments, we ran several univariate LMMs for each candidate gene. As the response we used the anxiety measure, time spent in the bottom zone (of the novel arena). As predictors, we used treatment group (control and 0.3ppb), relative mRNA of the candidate genes (each candidate gene was normalized by *b-actin* expression and centered using the ‘*scale*’ function) and an interaction term between treatment group and relative mRNA. Family ID was used as a random effect to account for genetic differences. The interaction term was removed if non-significant and models were re-analyzed. The effects of paternal atrazine exposure on transcript number alone were additionally assessed using linear mixed effect model with treatment, sex and an interaction term. Family ID was used as a random effect and the interaction term was removed if non-significant and models were reanalyzed without it (results are presented in supplementary material).

Significant (or marginally non-significant) differences are presented in text with estimates and their 95% confidence intervals (CI). Confidence intervals were calculated using the ‘*confint*’ function. For reference, 95% CI of estimates that do not include zero are statistically significant. The full model outputs, including parameter coefficients (back transformed where appropriate), parameter standard errors (SE), test statistics and p-values are reported in the supplementary material (Tables B, C and D in S1 File). P-values were calculated using the ‘*lmerTest*’ package [102]. Descriptive statistics presented in the results text are ranges and/or means ± SE.

## Results

### Effect of paternal atrazine exposure on F1 behavioral traits

#### Bottom-dwelling time

**Fig 1:**
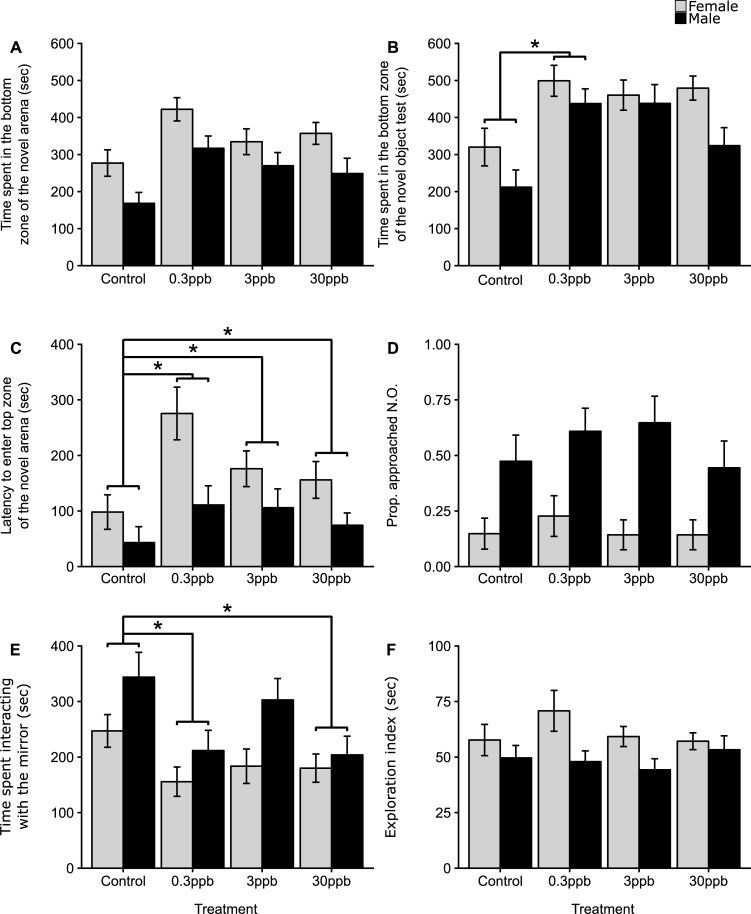
The effects of paternal atrazine exposure (at control [0ppb], 0.3ppb, 3ppb and 30ppb) on F1 behavioral traits. (A) the time spent in the bottom zone (s) during the novel arena test, (B) time spent in the bottom zone (s) during the novel object test, (C) the latency to enter the top zone (s) during the novel arena test, (D) the proportion of fish that approached the novel object test; (E) the time spent interacting with the mirror (s) and (F) exploration (s). Bars represent means, with error bars representing standard errors of the mean. An asterisk indicates a significant difference between controls and an atrazine treatment (p <0.05). For each sex and treatment, sample sizes ranged from n = 18–30. Total sample size for F1s were n = 190, with n = 3 families per treatment. See methods for sample sizes.

The time spent in the bottom zone in both the novel arena and novel object assay by F1 zebrafish ranged the full length of the assays from 0–600 s (Fig 1A and Fig 1B). We found no significant difference between controls and atrazine treatments in the time spent in the bottom zone during the novel arena test, though, visually, controls tended to spend less time in the bottom zone on average (Fig 1A). In the novel object test, however, F1 fish paternally exposed to 0.3ppb atrazine spent significantly more time in the bottom zone compared to F1 controls (Est. 180.19 s [13.18, 347.20 95% CI). Additionally, a marginally non-significant increase in bottom-dwelling time was observed in fish from the 3ppb treatment (Est. 163.90 s [−2.92, 330.71 95% CI]), but no significant differences were observed in offspring from the 30ppb treatment compared to controls (Fig 1B). Male fish spent less time in the bottom zone than females across both tests (novel arena test Est. −86.78 s [−128.30, −45.25 95% CI]; novel object test Est. −78.44 s [−137.31, −19.57 95% CI]) and averaged 253 ± 18 s and 355 ± 25 s in the bottom zone of the novel arena and novel object assay, respectively, whereas females spent an average of 344 ± 17 s and 437 ± 22 s, respectively.

#### Latency to enter the top zone

Paternal atrazine exposure significantly increased the latency to enter the top zone of the novel arena in all F1 atrazine treatment groups compared to controls (0.3ppb Est. 6.16 s [2.01, 18.73 95% CI]; 3ppb Est. 4.07 s [1.34, 12.30 95% CI]; 30ppb Est. 3.71 s [1.22, 11.25 95% CI]; Fig 1C). In addition, male fish, regardless of treatment, took significantly less time to enter the top zone (Est. −2.3 s [−2.32, −2.27 95% CI]), taking on average 84 ± 16 s, whereas females took an average of 171 ± 18 s to enter. There was no difference between treatments in the proportion of fish that approached the novel object (Fig 1D), but male fish were far more likely to approach than female fish (Est. 0.87 [0.76, 0.93 95% CI]), with on average 54% of males that approached compared to 16% of females (averaged across all treatment groups).

#### Interaction with a mirror

The time that F1 fish spent interacting with the mirror was significantly lower in the 0.3ppb treatment and 30ppb atrazine treatment compared to controls (0.3ppb Est. −110.63 s [−45.91, −3.35 95% CI]; 30ppb Est. −95.98 s [−31.72, −2.93 95% CI]; Fig 1E). Additionally, F1 fish from the 3ppb treatment exhibited a marginally non-significant decrease compared to control fish (3ppb Est. −56.32 s [−120.25, 7.61 95% CI]; Fig 1E). In general, male fish spent significantly more time engaging with the mirror than females (Est. 74.42 s [27.96, 120.89 95% CI]), spending an average of 264 ± 20 s interacting, whereas females spent on average 193 ± 14 s.

#### Exploration

Across all trials, the exploration index ranged from 15–177 s with a mean of 56 ± 2 s. There was no difference in exploratory behavior amongst the offspring of atrazine treated males compared to the offspring of control males (Fig 1F); however, male F1s were more exploratory than female F1s (Est. −12.43 s [−20.77, −4.09 95% CI]) and had an exploratory index mean of 49 ± 3 s, whereas females had a mean of 61 ± 3 s.

#### Activity

**Fig 2:**
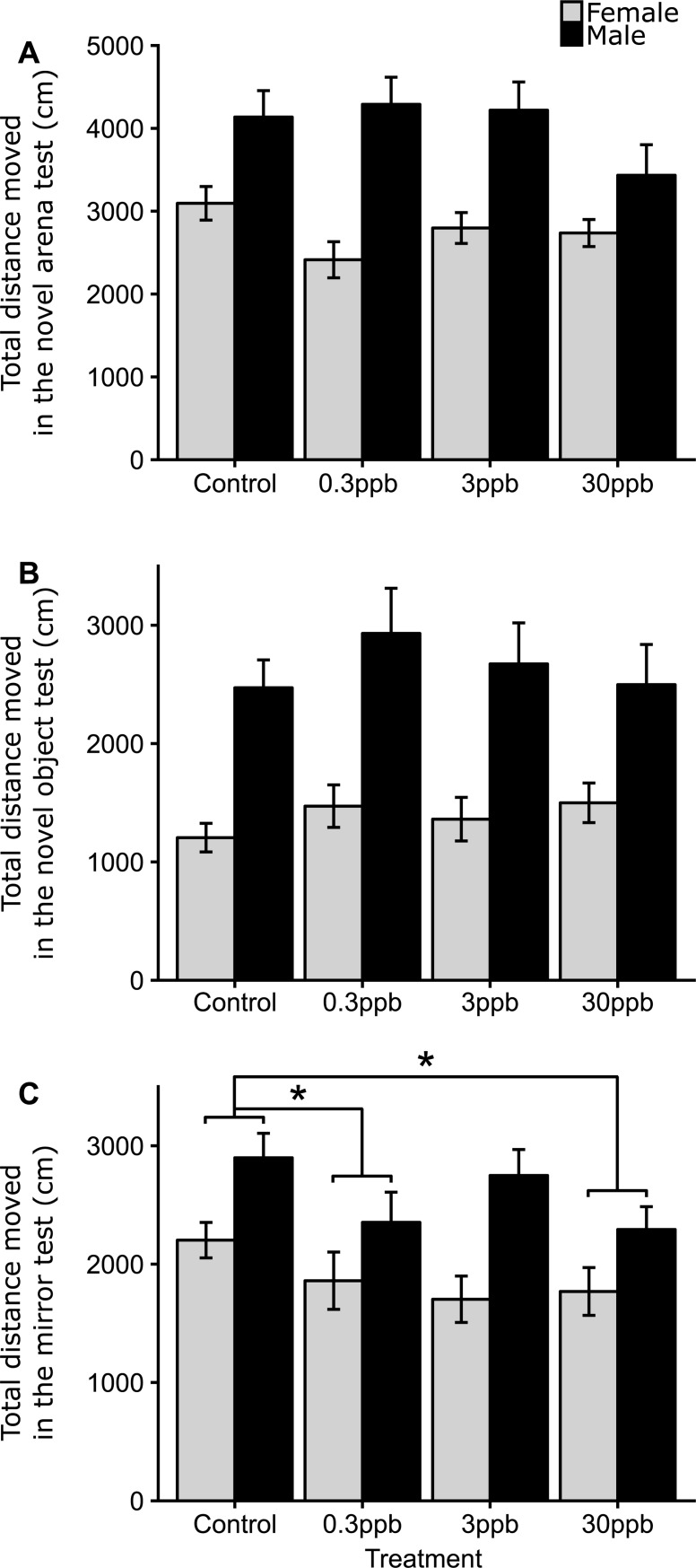
The effects of paternal atrazine exposure (at control [0ppb], 0.3ppb, 3ppb and 30ppb) on F1 activity, measured by the total distance moved (cm). (A) during the novel arena test, (B) the novel object test, (C) and during the mirror test. Bars represent means, with error bars representing standard errors of the mean. An asterisk indicates a significant difference between controls and an atrazine treatment (p <0.05). For each sex and treatment, sample sizes ranged from n = 18–30. Total sample size for F1s were n = 190, with n = 3 families per treatment. See methods for sample sizes.

During both the novel arena and novel object test, no significant differences in activity were observed across any of the treatment groups compared to controls (Fig 2A and Fig 2B). In contrast, offspring from the 0.3ppb and 30ppb treatment groups travelled significantly less than offspring from control males during the mirror test (0.3ppb Est. −110.63 cm [−175.35, −45.91 95% CI] and 30ppb Est. −95.98 cm [−160.24, −31.72 95% CI]; Fig 2C). Offspring from the 3ppb treatment also travelled less than control offspring, but this observation was marginally non-significant (Est. −56.32 cm [−120.25, 7.61 95% CI]). Male offspring were more active than female offspring, and travelled a greater total distance during the novel arena test (Est. 1255.64 cm [892.14, 1619.14 95% CI]), the novel object test (Est. 1257.18 cm [917.76, 1596.60 95% CI]) and during the mirror test (Est. 689.30 [393.78, 984.89 95% CI]). Overall, males travelled on average 4044 ± 170 cm, 2660 ± 167 cm and 2569 ± 115 cm, in the novel arena test, novel object test and the mirror test, respectively. Whereas females travelled 2780 ± 97 cm, 1381 ± 82 cm and 1881 ± 99, in the same tests.

### Effects of atrazine on mRNA transcript number

**Fig 3:**
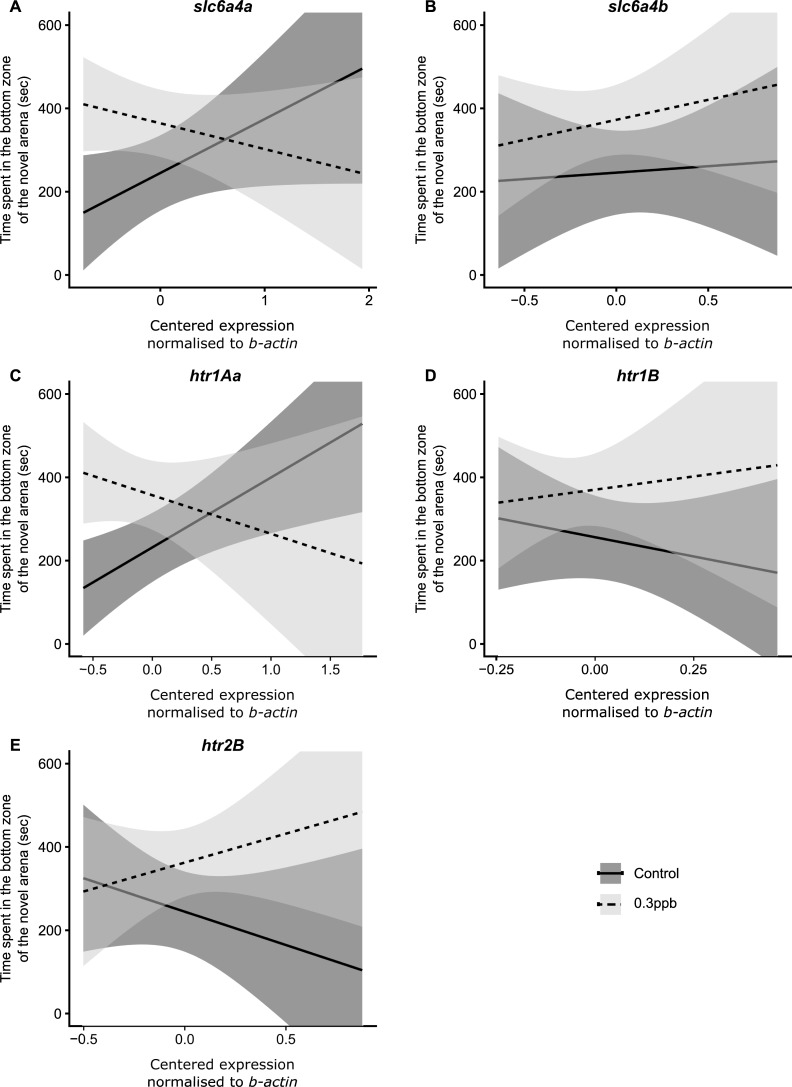
The relationship between time spent in the bottom zone of the novel arena (s) and relative mRNA levels of candidate genes. (A) *slc6a4a*, (B) *slc6a4b*, (C) *htr1Aa*, (D) *htr1B* and (E) *htr2B* of F1 offspring from control (solid line) and 0.3ppb atrazine treated (dashed line) males, normalized to b-actin and then centered (from whole-brain samples) and their 95% CI (shaded areas). Sample sizes for control offspring were n = 22 (males = 10; females = 12; progeny per family = 6–8; families n = 3), and for 0.3ppb offspring were n = 24 (males = 12; females = 12; progeny per family = 8; families n = 3).

Paternal atrazine exposure significantly altered the relationship between the time spent in the bottom zone of the novel arena (bottom-dwelling) and *slc6a4a* mRNA transcript number (Est. −191.46 s [−355.69, −27.23 95% CI]; Fig 3A). Bottom-dwelling behavior amongst F1 controls increased positively with increased *slc6a4a* mRNA transcript number whereas the opposite pattern was observed amongst F1 fish paternally exposed to 0.3ppb atrazine (Fig 3A). In contrast, no significant relationship was observed between bottom-dwelling and *slc6a4b* mRNA transcript number (Fig 3B). The relationship between bottom-dwelling behavior and *htr1Aa* mRNA was significantly altered between treatment groups (Est. −260.37 s [−465.39, −55.35 95% CI]; Fig 3C). *htr1Aa* mRNA transcript levels of control F1 fish increased with increased time spent in the bottom zone, whereas transcript levels of atrazine treated F1s decreased with increased time spent in the bottom zone (Fig 3C). There was no significant relationship between bottom-dwelling in the novel arena and either *htr1B* and *htr2B* mRNA transcript number (Fig 3D and Fig 3E, respectively).

## Discussion

We show that paternal exposure to the herbicide atrazine, during juvenile development, significantly influences a variety of behavioral traits of unexposed F1 offspring at adulthood (with many effects occurring in a non-dose dependent manner), including: bottom-dwelling time (at 0.3ppb, albeit non-significantly in the novel arena test), latency to enter the top zone of the arena (at 0.3ppb, 3ppb and 30ppb), interactions with a mirror (at 0.3ppb and 30ppb) and activity (at 0.3ppb and 30ppb during the aggression assay). Importantly, these effects are observed at environmentally relevant concentrations (0.3ppb is a typical low dose and 30ppb is a typical high dose likely to be encountered in the environment [35]). This study also shows that paternal atrazine exposure at 0.3ppb is involved in the disruption of some aspects of the serotonergic system, in particular, paternal atrazine exposure appears to have altered the trend between time spent in the bottom zone (a measure of anxiety) and *slc6a4a* mRNA and *htr1Aa* mRNA transcript number between treatments. Together, these results suggest that behavioral changes and underlying mRNA transcript difference might be transferred transgenerationally.

### Effects of paternal atrazine on F1 behavior

The trend of increased time spent in the bottom zone in the novel arena (at 0.3ppb) and the significant increase observed in time spent in the bottom zone of the novel object test (at 0.3ppb) suggest changes in anxiety due to low-dose paternal atrazine exposure. These differences are in line with previous work in fishes [103] and in rodents [54,60,104]. But the lack of substantial differences, when compared to the previous studies, may be due to the lower concentrations used and the intergenerational nature of this study design, as the aforementioned studies (exposed at concentrations up to 3000ppb in fish and up to 250 mg/kg in rodents) were either direct or prenatal exposures.

Paternal exposure to atrazine significantly increased the latency to enter the top zone of the novel arena at all concentrations tested (0.3ppb, 3ppb and 30ppb), with effects appearing to be more pronounced in females than males. These behavioral differences indicate a non-monotonic response, with the greatest increase observed in offspring of parents exposed to the lowest concentration. Previous work suggests that the latency to enter the top zone is often taken as a measure of anxiety-like behavior (e.g. [4,25,88,89,93]). In an ecological setting, entering the top portion of the water column where little vegetation occurs likely carries a higher risk of avian predation and may increase conspicuousness of the individual to fish predators [105–107]. When placed in a novel environment (such as in the novel arena test) or confronted by an avian predator simulation above the tank (e.g. a bird silhouette or a black dot increasing in size, simulating an approaching bird), defensive behaviors such as bottom-dwelling are triggered, and then over time, the fish will begin to explore the higher portions of the tank [88,93,108,109]. If the highest portion of the tank is perceived to carry a higher risk by the zebrafish, then changes in latency observed from atrazine treated offspring might reflect a distortion in an individual’s ability to either assess risk, or a change in propensity to take risks, regardless of the risk entailed. Risk assessment of the environment is crucial during foraging and exploration [110]. Individuals that under assess risk, or are generally bolder, have increased susceptibility to predation [111,112]. Whereas over assessment or more anxious individuals may have increased long-term survival, but may have costs on other functions, such as the ability to forage which may then have negative consequences on growth and reproduction [113,114].

Paternal atrazine exposure was found to significantly decrease interactions with a mirror (a proxy for aggression) in male and female offspring from the 0.3ppb and 30ppb treatment. The lack of a significant decrease in aggression in the 3ppb treatment offspring (though the decrease was marginally non-significant) may be due to the non-monotonic nature of atrazine as found in other studies [3,115–118]. For instance, Shenoy et al. [3,68] found that male guppies prenatally exposed to 1 ppb were consistently less aggressive (within a mating context) than those prenatally exposed to 15 ppb. Changes in activity levels during the mirror test (but not in the other two behavioral assays) might also suggest that zebrafish are less willing to engage in aggressive bouts or perhaps are warier to engage, as has been found previously in male-male interactions in guppies [68]. Reductions in aggression are in line with similar studies measuring male-male aggression and male sexual displays after direct atrazine exposure [3,52,53,68]. In the wild, aggression in zebrafish is important for establishing dominance hierarchies, enabling the monopolization of foraging resources and the maintenance of territories for spawning sites [119]. In addition, more aggressive zebrafish males exhibit higher reproductive success [98]. The consequences of reduced aggression may therefore limit the ability to compete for access to foraging sites and limit the ability to maintain territories. Aggression is also important in females, as either sex can establish a dominance hierarchy [119,120].

The behavioral measures tested here are known to exhibit long-term repeatability in zebrafish, using the same setting and testing regime [91]. Thus, it may be possible that atrazine has the capacity to affect animal personality traits in zebrafish as it has been shown in crayfish [70]. Some EDCs such as 17α-ethinylestradiol have been shown to disrupt individual consistency (e.g. [18–21]) and behavioral responses between individuals as a result of EDC exposure (including atrazine) can be personality dependent [70]. Hence, the behavioral data shown here may not capture the full extent to which atrazine is capable of altering animal behavior and highlights that further work is needed to investigate if atrazine exposure in fish shares similar endpoints.

### Effects of paternal atrazine on mRNA transcription in F1 brains

Paternal atrazine exposure appears to have altered the relationship between *slc6a4a* and *htr1Aa* mRNA and time spent in the bottom zone (a measure of anxiety in zebrafish), suggesting possible serotonergic dysregulation (Fig 3). In both genes, mRNA transcript was found to increase positively with the amount of time spent in the bottom zone amongst controls, whereas amongst fish that were paternally exposed to 0.3ppb atrazine, decreased mRNA transcript was associated with reduced time spent in the bottom zone. In addition, it appears that the relationship between *hrt2B* mRNA and bottom-dwelling behavior between treatments followed a similar pattern to the one found at *slc6a4a* and *htr1Aa* mRNA, but high individual variation likely precluded the detection of a significant interactive effect. The results of this study support previous research associating atrazine exposure with disruption of some aspects of the serotonergic system [5,54,60,61]. For example, adult mice exposed to atrazine (at 3mg/kg) during gestation and lactation, via atrazine in the mothers drinking water, exhibited decreased 5-HT levels in the striatum (and females additionally exhibited decreases in the perirhinal cortex [60]. Another study found disparities in the metabolome (the entire metabolite profile) of atrazine exposed male mice, including tryptophan (precursor to 5-HT) and other metabolites important in normal 5-HT function (e.g. linoleic acid and α- linolenic acid), at concentrations >5mg/kg [61]. At higher doses, (>125 mg/kg; but not at concentrations tested between 0–25 mg/kg) atrazine increased the 5-HT metabolite, 5-hydroxyindoleacetic acid (5-HIAA) levels and altered 5-HT turnover in the brain [54].

Previous research has found that bottom-dwelling in the novel arena test is inversely correlated with 5-HT levels and drugs targeted to interfere with *slc6a4a* and *htr1Aa* protein products can alter this behavior [76,121]. Indeed, blocking 5-HT re-uptake with fluoxetine treatment, thereby increasing 5-HT accumulation in the synaptic cleft, produces an anxiolytic effect in the novel arena test [76]. Furthermore, blocking 5-HT from binding to HT1A receptor (HTR1AR) on post synaptic membranes, using busporine (an agonist of the pre-synapse and antagonist on the post synapse) or WAY 100 635 (antagonist at both pre and post synapse), is associated with producing an anxiolytic effect in zebrafish [76]. The up-regulation of *slc6a4a*, *htr1Aa* and *htr1B* mRNA transcripts by the element selenium (in the dietary form of selenomethionine) is associated with increased bottom-dwelling behavior in zebrafish [122]. These results support the hypothesis that *slc6a4a* and *htr1Aa* (and potentially *htr1B*) gene expression are associated with anxiety in zebrafish.

While this study did not test for the effects of paternal atrazine exposure over developmental stages, it is possible that *slc6a4a* and *htr1Aa* mRNA disruption were present over ontogeny. Proper serotonergic function is critical during early life [123]. Indeed, disruption of HT1AR during adolescence in mice is also enough to sustain increased anxiety levels through to adulthood [74]. Likewise, HT1AR knockout mice exhibit increased anxiety at adulthood [124] and it appears that retaining proper function of HT1AR during development is critical for normal anxiety behavior as an adult [75]. Future studies could examine ontogenic features of atrazine exposure, anxiety and their relationship to *slc6a4a* and *htr1Aa* mRNA transcript levels.

The lack of a relationship at *slc6a4b* and *htr2B* mRNA transcript with bottom-dwelling might suggest that these genes are not as strongly associated with anxiety. The serotonergic system is a complex system that appears to govern a variety of behaviors other than anxiety such as aggression for which *slc6a4b* and *htr2B* mRNA transcripts and their protein products may be more involved in [9,125,126]. However, our results display a high level of variation, so it is possible that we did not have enough power to detect such an effect. Furthermore, while we did not find any significant interactive effect, we found it interesting that there was relatively minimal difference in the expression of bottom-dwelling at low transcript levels at *htr1B* and *htr2B* (and to some extent at *slc6a4b*) between treatments, but as mRNA transcript levels increased, the difference in bottom-dwelling behavior became more contrasting between the controls and treatment (Fig 3). In contrast, the expression of bottom-dwelling at low *slc6a4a* and *htr1Aa* mRNA transcript levels was disparate between treatments.

### Possible mechanisms underlying effects of atrazine on F1 progeny

We observed intergenerational effects of atrazine exposure that can only be attributable to the sperm of the exposed males, suggesting an underlying epigenetic mechanism. Epigenetic effects (i.e. DNA methylation, non-coding RNAs and histone modifications) are increasingly being studied [28,31,32,34,127–129] and it is known that exposure to strong environmental stressors (especially during early life) may leave epigenetic marks that, in turn, permanently alter the phenotype of the adult (see reviews; [128,130]). Studies suggest that the paternal DNA methylome is inherited in an unchanged state in zebrafish [131,132], as opposed to mammals (e.g. in humans and mice), where upon fertilization, both the maternal and paternal methylomes are erased and ‘re-programmed’, with only a small percentage (~5%) thought to escape this process [133–135]. Thus, in zebrafish, there is greater potential for transfer of environmental specific information. However, an epigenetic inheritance explanation requires transgenerational observations, i.e. the effect must be observed in the first generation where the germ line was not developmentally exposed [136,137], which would be the F2 generation in fish. However, previous research has shown that atrazine (at 25 mg/kg BW/day and 100 mg/kg/day) can induce transgenerational effects of disease in mice [138,139] and produce transgenerational reproductive defects (at 5ppb and 50ppb) in medaka [140]. Additionally, it appears that disease phenotypes can be exacerbated with each generation [139], suggesting that transgenerational effects on behavior are likely.

## Conclusions

In conclusion, we found that several aspects of progeny behavior were altered by paternal exposure to environmentally relevant concentrations of atrazine, thus representing intergenerational effects on behavior, though many of the effects on behavior were non dose-dependent. Moreover, some aspects of the serotonergic system were disrupted in the offspring, though given the high variation, further research is needed. Overall, these results add to the ecological consequences of environmental contaminants, most importantly, that further research may reveal that effects may be further propagated down the germ line.

## Supporting information

S1 File Supporting information. Details of supplementary methods including ethovision setup used for behavioral testing (Fig A in S1 File), primers used (Table A in S1 File), as well as supplementary results including treatment effects on transcript number (Fig B in S1 File) and model outputs (Tables B, C and D in S1 File).
(DOCX)

## References

[1] Clotfelter ED, Bell AM, Levering KR. The role of animal behaviour in the study of endocrine-disrupting chemicals. Anim Behav. 2004;68: 665–676.

[2] Söffker M, Tyler CR. Endocrine disrupting chemicals and sexual behaviors in fish–a critical review on effects and possible consequences. Crit Rev Toxicol. 2012;42: 653–668. 10.3109/10408444.2012.692114 22697575

[3] Shenoy K. Prenatal exposure to low doses of atrazine affects mating behaviors in male guppies. Horm Behav. 2014;66: 439–448. 10.1016/j.yhbeh.2014.07.002 25014197

[4] Volkova K, Caspillo NR, Porseryd T, Hallgren S, Dinnétz P, Porsch-Hällström I. Developmental exposure of zebrafish (*Danio rerio*) to 17α-ethinylestradiol affects non-reproductive behavior and fertility as adults, and increases anxiety in unexposed progeny. Horm Behav. 2015;73: 30–38. 10.1016/j.yhbeh.2015.05.014 26072466

[5] Wirbisky SE, Weber GJ, Sepúlveda MS, Xiao C, Cannon JR, Freeman JL. Developmental origins of neurotransmitter and transcriptome alterations in adult female zebrafish exposed to atrazine during embryogenesis. Toxicology. 2015;333: 156–167. 10.1016/j.tox.2015.04.016 25929836 PMC4471955

[6] Wirbisky SE, Weber GJ, Sepúlveda MS, Lin T-L, Jannasch AS, Freeman JL. An embryonic atrazine exposure results in reproductive dysfunction in adult zebrafish and morphological alterations in their offspring. Sci Rep. 2016;6: 21337 10.1038/srep21337 26891955 PMC4759560

[7] Wirbisky SE, Weber GJ, Schlotman KE, Sepúlveda MS, Freeman JL. Embryonic atrazine exposure alters zebrafish and human miRNAs associated with angiogenesis, cancer, and neurodevelopment. Food Chem Toxicol. 2016;98: 25–33. 10.1016/j.fct.2016.03.027 27046698 PMC5045766

[8] Wirbisky SE, Sepúlveda MS, Weber GJ, Jannasch AS, Horzmann KA, Freeman JL. Embryonic atrazine exposure elicits alterations in genes associated with neuroendocrine function in adult male zebrafish. Toxicol Sci. 2016;153: 149–164. 10.1093/toxsci/kfw115 27413107 PMC5013880

[9] Filby AL, Paull GC, Hickmore TF, Tyler CR. Unravelling the neurophysiological basis of aggression in a fish model. BMC Genomics. 2010;11: 498 10.1186/1471-2164-11-498 20846403 PMC2996994

[10] Thörnqvist P-O, Höglund E, Winberg S. Natural selection constrain personality and brain gene expression differences in Atlantic salmon (*Salmo salar*). J Exp Biol. 2015; 1077–1083. 10.1242/jeb.114314 25722007

[11] Whitfield CW, Cziko A-M, Robinson GE. Gene expression profiles in the brain predict behavior in individual honey bees. Science. 2003;302: 296–299. 10.1126/science.1086807 14551438

[12] Wiese A-S, Needham EK, Noer CL, Balsby TJS, Dabelsteen T, Pakkenberg B. The number of neurons in specific amygdala regions is associated with boldness in mink: a study in animal personality. Brain Struct Funct. 2018;223: 1989–1998. 10.1007/s00429-018-1606-4 29318377

[13] Diamanti-Kandarakis E, Bourguignon J-P, Giudice LC, Hauser R, Prins GS, Soto AM, et al Endocrine-disrupting chemicals: an Endocrine Society scientific statement. Endocr Rev. 2009;30: 293–342. 10.1210/er.2009-0002 19502515 PMC2726844

[14] Hotchkiss AK, Rider CV, Blystone CR, Wilson VS, Hartig PC, Ankley GT, et al Fifteen years after “Wingspread”—environmental endocrine disrupters and human and wildlife health: where we are today and where we need to go. Toxicol Sci. 2008;105: 235–259. 10.1093/toxsci/kfn030 18281716 PMC2721670

[15] Vandenberg LN, Colborn T, Hayes TB, Heindel JJ, Jacobs DR Jr, Lee D-H, et al Hormones and endocrine-disrupting chemicals: low-dose effects and nonmonotonic dose responses. Endocr Rev. 2012;33: 378–455. 10.1210/er.2011-1050 22419778 PMC3365860

[16] Bertram MG, Saaristo M, Ecker TE, Baumgartner JB, Wong B. An androgenic endocrine disruptor alters male mating behavior in the guppy (*Poecilia reticulata*). Behav Ecol. 2018; 1255–1263.

[17] Bertram MG, Saaristo M, Martin JM, Ecker TE, Michelangeli M, Johnstone CP, et al Field-realistic exposure to the androgenic endocrine disruptor 17β-trenbolone alters ecologically important behaviours in female fish across multiple contexts. Environ Pollut. 2018;243: 900–911. 10.1016/j.envpol.2018.09.044 30245452

[18] Dzieweczynski TL. Short-term exposure to an endocrine disruptor affects behavioural consistency in male threespine stickleback. Aquat Toxicol. 2011;105: 681–687. 10.1016/j.aquatox.2011.09.010 21975189

[19] Dzieweczynski TL, Campbell BA, Marks JM, Logan B. Acute exposure to 17α-ethinylestradiol alters boldness behavioral syndrome in female Siamese fighting fish. Horm Behav. 2014;66: 577–584. 10.1016/j.yhbeh.2014.08.005 25161058

[20] Dzieweczynski TL, Hebert OL. The effects of short-term exposure to an endocrine disrupter on behavioral consistency in male juvenile and adult Siamese fighting fish. Arch Environ Contam Toxicol. 2013;64: 316–326. 10.1007/s00244-012-9820-1 23073845

[21] Dzieweczynski TL, Kane JL, Campbell BA, Lavin LE. Fluoxetine exposure impacts boldness in female Siamese fighting fish, *Betta splendens*. Ecotoxicology. 2016;25: 69–79. 10.1007/s10646-015-1568-8 26462842

[22] Hebert OL, Lavin LE, Marks JM, Dzieweczynski TL. The effects of 17α-ethinyloestradiol on boldness and its relationship to decision making in male Siamese fighting fish. Anim Behav. 2014;87: 203–212.

[23] Lagesson A, Saaristo M, Brodin T, Fick J, Klaminder J, Martin J, et al Fish on steroids: Temperature-dependent effects of 17β-trenbolone on predator escape, boldness, and exploratory behaviors. Environ Pollut. 2019;245: 243–252. 10.1016/j.envpol.2018.10.116 30423539

[24] Porseryd T, Kellner M, Caspillo NR, Volkova K, Elabbas L, Ullah S, et al Combinatory effects of low concentrations of 17α-etinylestradiol and citalopram on non-reproductive behavior in adult zebrafish (*Danio rerio*). Aquat Toxicol. 2017;193: 9–17. 10.1016/j.aquatox.2017.10.001 29017090

[25] Volkova K, Caspillo NR, Porseryd T, Hallgren S, Dinnetz P, Olsén H, et al Transgenerational effects of 17α-ethinyl estradiol on anxiety behavior in the guppy, (*Poecilia reticulata*). Gen Comp Endocrinol. 2015;223: 66–72. 10.1016/j.ygcen.2015.09.027 26431611

[26] Bonduriansky R, Crean AJ, Day T. The implications of nongenetic inheritance for evolution in changing environments. Evol Appl. 2012;5: 192–201. 10.1111/j.1752-4571.2011.00213.x 25568041 PMC3353344

[27] Crews D, Gore AC, Hsu TS, Dangleben NL, Spinetta M, Schallert T, et al Transgenerational epigenetic imprints on mate preference. Proc Natl Acad Sci. 2007;104: 5942–5946. 10.1073/pnas.0610410104 17389367 PMC1851596

[28] O’Dea RE, Noble DW, Johnson SL, Hesselson D, Nakagawa S. The role of non-genetic inheritance in evolutionary rescue: epigenetic buffering, heritable bet hedging and epigenetic traps. Environ Epigenetics. 2016;2. 10.1093/eep/dvv014 PMC5804513 29492283

[29] Wolstenholme JT, Edwards M, Shetty SR, Gatewood JD, Taylor JA, Rissman EF, et al Gestational exposure to bisphenol a produces transgenerational changes in behaviors and gene expression. Endocrinology. 2012;153: 3828–3838. 10.1210/en.2012-1195 22707478 PMC3404345

[30] Inagaki T, Smith N, Sherva K, Ramakrishnan S. Cross-generational effects of parental low dose BPA exposure on the Gonadotropin-Releasing Hormone3 system and larval behavior in medaka (*Oryzias latipes*). Neurotoxicology. 2016;57: 163–173. 10.1016/j.neuro.2016.09.021 27713093

[31] Badyaev AV, Uller T. Parental effects in ecology and evolution: mechanisms, processes and implications. Philos Trans R Soc Lond B Biol Sci. 2009;364: 1169–1177. 10.1098/rstb.2008.0302 19324619 PMC2666689

[32] Curley JP, Mashoodh R, Champagne FA. Epigenetics and the origins of paternal effects. Horm Behav. 2011;59: 306–314. 10.1016/j.yhbeh.2010.06.018 20620140 PMC2975825

[33] Giesing ER, Suski CD, Warner RE, Bell AM. Female sticklebacks transfer information via eggs: effects of maternal experience with predators on offspring. Proc R Soc B Biol Sci. 2010;278: 1753–1759. 10.1098/rspb.2010.1819 PMC3081764 21068041

[34] Sopinka N, Capelle P, Semeniuk CA, Love OP. Glucocorticoids in fish eggs: variation, interactions with the environment, and the potential to shape offspring fitness. Physiol Biochem Zool. 2017;90: 15–33. 10.1086/689994 28051944

[35] Graymore M, Stagnitti F, Allinson G. Impacts of atrazine in aquatic ecosystems. Environ Int. 2001;26: 483–495. 10.1016/s0160-4120(01)00031-9 11485216

[36] Marshall J Dustin, Uller T. When is a maternal effect adaptive? Oikos. 2007;116: 1957–1963.

[37] Mousseau TA, Fox CW. The adaptive significance of maternal effects. Trends Ecol Evol. 1998;13: 403–407. 10.1016/s0169-5347(98)01472-4 21238360

[38] Nishi M, Horii-Hayashi N, Sasagawa T, Matsunaga W. Effects of early life stress on brain activity: implications from maternal separation model in rodents. Gen Comp Endocrinol. 2013;181: 306–309. 10.1016/j.ygcen.2012.09.024 23032077

[39] Nishi M, Horii-Hayashi N, Sasagawa T. Effects of early life adverse experiences on the brain: implications from maternal separation models in rodents. Front Neurosci. 2014;8: 166 10.3389/fnins.2014.00166 24987328 PMC4060417

[40] Wolf JB, Wade MJ. What are maternal effects (and what are they not)? Philos Trans R Soc Lond B Biol Sci. 2009;364: 1107–1115. 10.1098/rstb.2008.0238 19324615 PMC2666680

[41] Dias BG, Ressler KJ. Parental olfactory experience influences behavior and neural structure in subsequent generations. Nat Neurosci. 2014;17: 89–96. 10.1038/nn.3594 24292232 PMC3923835

[42] Immler S. The sperm factor: paternal impact beyond genes. Heredity. 2018;121: 239–247. 10.1038/s41437-018-0111-0 29959427 PMC6082889

[43] Zajitschek S, Hotzy C, Zajitschek F, Immler S. Short-term variation in sperm competition causes sperm-mediated epigenetic effects on early offspring performance in the zebrafish. Proc R Soc Lond B Biol Sci. 2014;281: 20140422. 10.1098/rspb.2014.0422 PMC4024299 24789902

[44] Anway MD, Cupp AS, Uzumcu M, Skinner MK. Epigenetic transgenerational actions of endocrine disruptors and male fertility. Science. 2005;308: 1466–1469. 10.1126/science.1108190 15933200 PMC11423801

[45] Mainwaring MC, Hartley IR. Hatching asynchrony and offspring sex influence the subsequent exploratory behaviour of zebra finches. Anim Behav. 2013;85: 77–81.

[46] Reddon AR. Parental effects on animal personality. Behav Ecol. 2011;23: 242–245.

[47] Rokka K, Pihlaja M, Siitari H, Soulsbury CD. Sex-specific differences in offspring personalities across the laying order in magpies *Pica pica*. Behav Processes. 2014;107: 79–87. 10.1016/j.beproc.2014.07.019 25111085

[48] Barr DB, Panuwet P, Nguyen JV, Udunka S, Needham LL. Assessing exposure to atrazine and its metabolites using biomonitoring. Environ Health Perspect. 2007;115: 1474–1478. 10.1289/ehp.10141 17938738 PMC2022667

[49] Solomon KR, Carr JA, Du Preez LH, Giesy JP, Kendall RJ, Smith EE, et al Effects of atrazine on fish, amphibians, and aquatic reptiles: a critical review. Crit Rev Toxicol. 2008;38: 721–772. 10.1080/10408440802116496 18941967

[50] Singh S, Kumar V, Chauhan A, Datta S, Wani AB, Singh N, et al Toxicity, degradation and analysis of the herbicide atrazine. Environ Chem Lett. 2018;16: 211–237.

[51] Rohr JR, McCoy KA. A qualitative meta-analysis reveals consistent effects of atrazine on freshwater fish and amphibians. Environ Health Perspect. 2009;118: 20–32. 10.1289/ehp.0901164 PMC2831963 20056568

[52] Hayes TB, Khoury V, Narayan A, Nazir M, Park A, Brown T, et al Atrazine induces complete feminization and chemical castration in male African clawed frogs (*Xenopus laevis*). Proc Natl Acad Sci. 2010; 200909519. 10.1073/pnas.0909519107 PMC2842049 20194757

[53] Hayes TB, Collins A, Lee M, Mendoza M, Noriega N, Stuart AA, et al Hermaphroditic, demasculinized frogs after exposure to the herbicide atrazine at low ecologically relevant doses. Proc Natl Acad Sci. 2002;99: 5476–5480. 10.1073/pnas.082121499 11960004 PMC122794

[54] Lin Z, Dodd CA, Filipov NM. Short-term atrazine exposure causes behavioral deficits and disrupts monoaminergic systems in male C57BL/6 mice. Neurotoxicol Teratol. 2013;39: 26–35. 10.1016/j.ntt.2013.06.002 23770127

[55] Horzmann KA, Reidenbach LS, Thanki DH, Winchester AE, Qualizza BA, Ryan GA, et al Embryonic atrazine exposure elicits proteomic, behavioral, and brain abnormalities with developmental time specific gene expression signatures. J Proteomics. 2018;186: 71–82. 10.1016/j.jprot.2018.07.006 30012420 PMC6193558

[56] Richter CA, Papoulias DM, Whyte JJ, Tillitt DE. Evaluation of potential mechanisms of atrazine‐induced reproductive impairment in fathead minnow (*Pimephales promelas*) and Japanese medaka (*Oryzias latipes*). Environ Toxicol Chem. 2016;35: 2230–2238. 10.1002/etc.3376 26792394

[57] Foradori CD, Hinds LR, Hanneman WH, Legare ME, Clay CM, Handa RJ. Atrazine inhibits pulsatile luteinizing hormone release without altering pituitary sensitivity to a gonadotropin-releasing hormone receptor agonist in female Wistar rats. Biol Reprod. 2009;81: 40–45. 10.1095/biolreprod.108.075713 19299313

[58] Foradori CD, Zimmerman AD, Hinds LR, Zuloaga KL, Breckenridge CB, Handa RJ. Atrazine inhibits pulsatile gonadotropin-releasing hormone (GnRH) release without altering GnRH messenger RNA or protein levels in the female rat. Biol Reprod. 2013;88: 9–1. 10.1095/biolreprod.112.102277 23197165

[59] Cooper RL, Laws SC, Das PC, Narotsky MG, Goldman JM, Lee Tyrey E, et al Atrazine and reproductive function: mode and mechanism of action studies. Birth Defects Res B Dev Reprod Toxicol. 2007;80: 98–112. 10.1002/bdrb.20110 17443714

[60] Lin Z, Dodd CA, Xiao S, Krishna S, Ye X, Filipov NM. Gestational and lactational exposure to atrazine via the drinking water causes specific behavioral deficits and selectively alters monoaminergic systems in C57BL/6 mouse dams, juvenile and adult offspring. Toxicol Sci. 2014;141: 90–102. 10.1093/toxsci/kfu107 24913803 PMC4184358

[61] Lin Z, Roede JR, He C, Jones DP, Filipov NM. Short-term oral atrazine exposure alters the plasma metabolome of male C57BL/6 mice and disrupts α-linolenate, tryptophan, tyrosine and other major metabolic pathways. Toxicology. 2014;326: 130–141. 10.1016/j.tox.2014.11.001 25445803

[62] Rodríguez VM, Limón-Pacheco JH, Mendoza-Trejo MS, González-Gallardo A, Hernández-Plata I, Giordano M. Repeated exposure to the herbicide atrazine alters locomotor activity and the nigrostriatal dopaminergic system of the albino rat. Neurotoxicology. 2013;34: 82–94. 10.1016/j.neuro.2012.10.012 23123945

[63] Sanderson JT, Boerma J, Lansbergen GW, van den Berg M. Induction and inhibition of aromatase (CYP19) activity by various classes of pesticides in H295R human adrenocortical carcinoma cells. Toxicol Appl Pharmacol. 2002;182: 44–54. 10.1006/taap.2002.9420 12127262

[64] Abarikwu S, Farombi E, Kashyap M, Pant A. Atrazine induces transcriptional changes in marker genes associated with steroidogenesis in primary cultures of rat Leydig cells. Toxicol In Vitro. 2011;25: 1588–1595. 10.1016/j.tiv.2011.06.002 21693180

[65] Kucka M, Pogrmic-Majkic K, Fa S, Stojilkovic SS, Kovacevic R. Atrazine acts as an endocrine disrupter by inhibiting cAMP-specific phosphodiesterase-4. Toxicol Appl Pharmacol. 2012;265: 19–26. 10.1016/j.taap.2012.09.019 23022511 PMC4181665

[66] Roberge M, Hakk H, Larsen G. Atrazine is a competitive inhibitor of phosphodiesterase but does not affect the estrogen receptor. Toxicol Lett. 2004;154: 61–68. 10.1016/j.toxlet.2004.07.005 15475179

[67] Li J, Li X, Bi H, Ma K, Li B. Developmental exposure to atrazine impairs spatial memory and downregulates the hippocampal D1 dopamine receptor and cAMP-dependent signaling pathway in rats. Int J Mol Sci. 2018;19: 2241. 10.3390/ijms19082241 PMC6121906 30065202

[68] Shenoy K. Environmentally realistic exposure to the herbicide atrazine alters some sexually selected traits in male guppies. PLoS One. 2012;7: e30611 10.1371/journal.pone.0030611 22312428 PMC3270011

[69] Schmidel AJ, Assmann KL, Werlang CC, Bertoncello KT, Francescon F, Rambo CL, et al Subchronic atrazine exposure changes defensive behaviour profile and disrupts brain acetylcholinesterase activity of zebrafish. Neurotoxicol Teratol. 2014;44: 62–69. 10.1016/j.ntt.2014.05.006 24893294

[70] Steele AN, Moore PA. Express yourself: Individuals with bold personalities exhibit increased behavioral sensitivity to dynamic herbicide exposure. Ecotoxicol Environ Saf. 2019;179: 272–281. 10.1016/j.ecoenv.2019.04.069 31059994

[71] Bardullas U, Giordano M, Rodríguez VM. Chronic atrazine exposure causes disruption of the spontaneous locomotor activity and alters the striatal dopaminergic system of the male Sprague–Dawley rat. Neurotoxicol Teratol. 2011;33: 263–272. 10.1016/j.ntt.2010.09.001 20850525

[72] del Carmen Alvarez M, Fuiman LA. Environmental levels of atrazine and its degradation products impair survival skills and growth of red drum larvae. Aquat Toxicol. 2005;74: 229–241. 10.1016/j.aquatox.2005.05.014 16009435

[73] Weber GJ, Sepúlveda MS, Peterson SM, Lewis SS, Freeman JL. Transcriptome alterations following developmental atrazine exposure in zebrafish are associated with disruption of neuroendocrine and reproductive system function, cell cycle, and carcinogenesis. Toxicol Sci. 2013;132: 458–466. 10.1093/toxsci/kft017 23358194 PMC3595526

[74] Garcia-Garcia AL, Meng Q, Richardson-Jones J, Dranovsky A, Leonardo ED. Disruption of 5-HT1A function in adolescence but not early adulthood leads to sustained increases of anxiety. Neuroscience. 2016;321: 210–221. 10.1016/j.neuroscience.2015.05.076 26049143 PMC4669240

[75] Gross C, Zhuang X, Stark K, Ramboz S, Oosting R, Kirby L, et al Serotonin 1A receptor acts during development to establish normal anxiety-like behaviour in the adult. Nature. 2002;416: 396–400. 10.1038/416396a 11919622

[76] Maximino C, Puty B, Benzecry R, Araújo J, Lima MG, Batista E de JO, et al Role of serotonin in zebrafish (*Danio rerio*) anxiety: relationship with serotonin levels and effect of buspirone, WAY 100635, SB 224289, fluoxetine and para-chlorophenylalanine (pCPA) in two behavioral models. Neuropharmacology. 2013;71: 83–97. 10.1016/j.neuropharm.2013.03.006 23541719

[77] Norton WH, Folchert A, Bally-Cuif L. Comparative analysis of serotonin receptor (HTR1A/HTR1B families) and transporter (*slc6a4a/b*) gene expression in the zebrafish brain. J Comp Neurol. 2008;511: 521–542. 10.1002/cne.21831 18839395

[78] Piñeyro G, Blier P. Autoregulation of serotonin neurons: role in antidepressant drug action. Pharmacol Rev. 1999;51: 533–591. 10471417

[79] Wang Y, Takai R, Yoshioka H, Shirabe K. Characterization and expression of serotonin transporter genes in zebrafish. Tohoku J Exp Med. 2006;208: 267–274. 10.1620/tjem.208.267 16498236

[80] Craig IW, Halton KE. Genetics of human aggressive behaviour. Hum Genet. 2009;126: 101–113. 10.1007/s00439-009-0695-9 19506905

[81] Holtmann B, Grosser S, Lagisz M, Johnson S, Santos E, Lara C, et al Population differentiation and behavioural association of the two `personality’ genes DRD4 and SERT in dunnocks (*Prunella modularis*). Mol Ecol. 2016;25: 706–722. 10.1111/mec.13514 26669286

[82] Miller-Butterworth CM, Kaplan JR, Barmada MM, Manuck SB, Ferrell RE. The serotonin transporter: sequence variation in *Macaca fascicularis* and its relationship to dominance. Behav Genet. 2007;37: 678–696. 10.1007/s10519-007-9162-3 17605101

[83] Müller JC, Partecke J, Hatchwell BJ, Gaston KJ, Evans KL. Candidate gene polymorphisms for behavioural adaptations during urbanization in blackbirds. Mol Ecol. 2013;22: 3629–3637. 10.1111/mec.12288 23495914

[84] Johnson SL, Zellhuber-McMillan S, Gillum J, Dunleavy J, Evans JP, Nakagawa S, et al Evidence that fertility trades off with early offspring fitness as males age. Proc R Soc B Biol Sci. 2018;285. 10.1098/rspb.2017.2174 PMC5805930 29367392

[85] Lamb SD. Early exposure to atrazine in zebrafish: intergenerational effects and animal personality. Master’s Thesis, University of Otago. 2018.

[86] Takahashi H. Juvenile hermaphroditism in the zebrafish, *Brachydanio rerio*. Bull Fac Fish Hokkaido Univ. 1977;28: 57–65.

[87] Uchida D, Yamashita M, Kitano T, Iguchi T. Oocyte apoptosis during the transition from ovary-like tissue to testes during sex differentiation of juvenile zebrafish. J Exp Biol. 2002;205: 711–718. 11914381 10.1242/jeb.205.6.711

[88] Egan RJ, Bergner CL, Hart PC, Cachat JM, Canavello PR, Elegante MF, et al Understanding behavioral and physiological phenotypes of stress and anxiety in zebrafish. Behav Brain Res. 2009;205: 38–44. 10.1016/j.bbr.2009.06.022 19540270 PMC2922906

[89] Maximino C, de Brito TM, da Silva Batista AW, Herculano AM, Morato S, Gouveia A Jr. Measuring anxiety in zebrafish: a critical review. Behav Brain Res. 2010;214: 157–171. 10.1016/j.bbr.2010.05.031 20510300

[90] Way GP, Ruhl N, Snekser JL, Kiesel AL, McRobert SP. A comparison of methodologies to test aggression in zebrafish. Zebrafish. 2015;12: 144–151. 10.1089/zeb.2014.1025 25621988

[91] Thomson HR. Consistency of behaviours over time and context in zebrafish. Master’s Thesis, University of Otago. 2017.

[92] Noldus LP, Spink AJ, Tegelenbosch RA. EthoVision: a versatile video tracking system for automation of behavioral experiments. Behav Res Methods Instrum Comput. 2001;33: 398–414. 10.3758/bf03195394 11591072

[93] Maximino C, Benzecry R, Oliveira KRM, Batista E de JO, Herculano AM, Rosemberg DB, et al A comparison of the light/dark and novel tank tests in zebrafish. Behaviour. 2012;149: 1099–1123.

[94] Wright D, Nakamichi R, Krause J, Butlin RK. QTL analysis of behavioral and morphological differentiation between wild and laboratory zebrafish (*Danio rerio*). Behav Genet. 2006;36: 271–284. 10.1007/s10519-005-9029-4 16408248

[95] Wright D, Rimmer LB, Pritchard VL, Butlin R, Krause J. Inter and intra-population variation in shoaling and boldness in the zebrafish (*Danio rerio*). Naturwissenschaften. 2003;90: 374–377. 10.1007/s00114-003-0443-2 12955228

[96] Ogwang SP. Inspection of a novel object by wild and laboratory zebrafish (*Danio rerio*). Master’s Thesis, University of Bergen. 2017.

[97] Ariyomo TO, Carter M, Watt PJ. Heritability of boldness and aggressiveness in the zebrafish. Behav Genet. 2013;43: 161–167. 10.1007/s10519-013-9585-y 23354973

[98] Ariyomo TO, Watt PJ. The effect of variation in boldness and aggressiveness on the reproductive success of zebrafish. Anim Behav. 2012;83: 41–46.

[99] Oliveira RF, Carneiro LA, Canário AV. Behavioural endocrinology: no hormonal response in tied fights. Nature. 2005;437: 207–208. 10.1038/437207a 16148924

[100] R Core Team RF for SC Vienna, Austria. R: A language and environment for statistical computing. 2018. Available: https://www.R-project.org/

[101] Bates D, Mächler M, Bolker B, Walker S. Fitting linear mixed-effects models using lme4. J Stat Softw. 2014;67: 1–48.

[102] Kuznetsova A, Brockhoff PB, Christensen RHB. lmerTest package: tests in linear mixed effects models. J Stat Softw. 2017;82 10.18637/jss.v082.i02

[103] Steinberg CE, Lorenz R, Spieser OH. Effects of atrazine on swimming behavior of zebrafish, *Brachydanio rerio*. Water Res. 1995;29: 981–985.

[104] Walters JL, Lansdell TA, Lookingland KJ, Baker LE. The effects of gestational and chronic atrazine exposure on motor behaviors and striatal dopamine in male Sprague-Dawley rats. Toxicol Appl Pharmacol. 2015;289: 185–192. 10.1016/j.taap.2015.09.026 26440580 PMC5113295

[105] Pink M, Abrahams MV. In shallow water ecosytems the abiotic environment is more important than prey abundance for foraging terns. Environ Biol Fishes. 2018;101: 355–362.

[106] Rypel AL, Layman CA, Arrington DA. Water depth modifies relative predation risk for a motile fish taxon in Bahamian tidal creeks. Estuaries Coasts. 2007;30: 518–525.

[107] Werner EE, Gilliam JF, Hall DJ, Mittelbach GG. An experimental test of the effects of predation risk on habitat use in fish. Ecology. 1983;64: 1540–1548.

[108] Luca RM, Gerlai R. Animated bird silhouette above the tank: acute alcohol diminishes fear responses in zebrafish. Behav Brain Res. 2012;229: 194–201. 10.1016/j.bbr.2012.01.021 22266470 PMC3293988

[109] Luca RM, Gerlai R. In search of optimal fear inducing stimuli: differential behavioral responses to computer animated images in zebrafish. Behav Brain Res. 2012;226: 66–76. 10.1016/j.bbr.2011.09.001 21920389 PMC3203217

[110] Pitcher T, Lang S, Turner J. A risk-balancing trade off between foraging rewards and predation hazard in a shoaling fish. Behav Ecol Sociobiol. 1988;22: 225–228.

[111] MacPherson B, Mashayekhi M, Gras R, Scott R. Exploring the connection between emergent animal personality and fitness using a novel individual-based model and decision tree approach. Ecol Inform. 2017;40: 81–92.

[112] Smith BR, Blumstein DT. Fitness consequences of personality: a meta-analysis. Behav Ecol. 2008;19: 448–455.

[113] Biro PA, Stamps JA. Are animal personality traits linked to life-history productivity? Trends Ecol Evol. 2008;23: 361–368. 10.1016/j.tree.2008.04.003 18501468

[114] Wolf M, Van Doorn GS, Leimar O, Weissing FJ. Life-history trade-offs favour the evolution of animal personalities. Nature. 2007;447: 581 10.1038/nature05835 17538618

[115] Brodeur JC, Sassone A, Hermida GN, Codugnello N. Environmentally-relevant concentrations of atrazine induce non-monotonic acceleration of developmental rate and increased size at metamorphosis in *Rhinella arenarum* tadpoles. Ecotoxicol Environ Saf. 2013;92: 10–17. 10.1016/j.ecoenv.2013.01.019 23499184

[116] Marcus SR, Fiumera AC. Atrazine exposure affects longevity, development time and body size in *Drosophila melanogaster*. J Insect Physiol. 2016;91: 18–25. 10.1016/j.jinsphys.2016.06.006 27317622 PMC4969214

[117] McCallum ML, Matlock M, Treas J, Safi B, Sanson W, McCallum JL. Endocrine disruption of sexual selection by an estrogenic herbicide in the mealworm beetle (*Tenebrio molitor*). Ecotoxicology. 2013;22: 1461–1466. 10.1007/s10646-013-1132-3 24085605

[118] Riffle BW, Klinefelter GR, Cooper RL, Winnik WM, Swank A, Jayaraman S, et al Novel molecular events associated with altered steroidogenesis induced by exposure to atrazine in the intact and castrate male rat. Reprod Toxicol. 2014;47: 59–69. 10.1016/j.reprotox.2014.05.008 24887032

[119] Spence R, Gerlach G, Lawrence C, Smith C. The behaviour and ecology of the zebrafish, *Danio rerio*. Biol Rev. 2008;83: 13–34. 10.1111/j.1469-185X.2007.00030.x 18093234

[120] Grant JW, Kramer DL. Temporal clumping of food arrival reduces its monopolization and defence by zebrafish, *Brachydanio rerio*. Anim Behav. 1992;44: 101–110.

[121] Herculano AM, Maximino C. Serotonergic modulation of zebrafish behavior: towards a paradox. Prog Neuropsychopharmacol Biol Psychiatry. 2014;55: 50–66. 10.1016/j.pnpbp.2014.03.008 24681196

[122] Attaran A, Salahinejad A, Crane AL, Niyogi S, Chivers DP. Chronic exposure to dietary selenomethionine dysregulates the genes involved in serotonergic neurotransmission and alters social and antipredator behaviours in zebrafish (*Danio rerio*). Environ Pollut. 2019;246: 837–844. 10.1016/j.envpol.2018.12.090 30623840

[123] Airhart MJ, Lee DH, Wilson TD, Miller BE, Miller MN, Skalko RG, et al Adverse effects of serotonin depletion in developing zebrafish. Neurotoxicol Teratol. 2012;34: 152–160. 10.1016/j.ntt.2011.08.008 21893190

[124] Zhuang X, Gross C, Santarelli L, Compan V, Trillat A-C, Hen R. Altered emotional states in knockout mice lacking 5-HT1A or 5-HT1B receptors. Neuropsychopharmacology. 1999;21: 52S 10.1016/S0893-133X(99)00047-0 10432489

[125] Theodoridi A, Tsalafouta A, Pavlidis M. Acute exposure to fluoxetine alters aggressive behavior of zebrafish and expression of genes involved in serotonergic system regulation. Front Neurosci. 2017;11: 223 10.3389/fnins.2017.00223 28487628 PMC5403945

[126] Rosengren M, Thörnqvist P-O, Winberg S, Sundell K. The brain-gut axis of fish: rainbow trout with low and high cortisol response show innate differences in intestinal integrity and brain gene expression. Gen Comp Endocrinol. 2018;257: 235–245. 10.1016/j.ygcen.2017.09.020 28947388

[127] Casas E, Vavouri T. Sperm epigenomics: challenges and opportunities. Front Genet. 2014;5: 330 10.3389/fgene.2014.00330 25278962 PMC4166955

[128] Jablonka E, Raz G. Transgenerational epigenetic inheritance: prevalence, mechanisms, and implications for the study of heredity and evolution. Q Rev Biol. 2009;84: 131–176. 10.1086/598822 19606595

[129] Rando OJ. Intergenerational transfer of epigenetic information in sperm. Cold Spring Harb Perspect Med. 2016; a022988 10.1101/cshperspect.a022988 26801897 PMC4852801

[130] Barker D. The developmental origins of adult disease. J Am Coll Nutr. 2004;23: 588S–595S. 10.1080/07315724.2004.10719428 15640511

[131] Jiang L, Zhang J, Wang J-J, Wang L, Zhang L, Li G, et al Sperm, but not oocyte, DNA methylome is inherited by zebrafish early embryos. Cell. 2013;153: 773–784. 10.1016/j.cell.2013.04.041 23663777 PMC4081501

[132] Potok ME, Nix DA, Parnell TJ, Cairns BR. Reprogramming the maternal zebrafish genome after fertilization to match the paternal methylation pattern. Cell. 2013;153: 759–772. 10.1016/j.cell.2013.04.030 23663776 PMC4030421

[133] Hackett JA, Surani MA. Beyond DNA: programming and inheritance of parental methylomes. Cell. 2013;153: 737–739. 10.1016/j.cell.2013.04.044 23663772 PMC4338575

[134] Lees-Murdock DJ, Walsh CP. DNA methylation reprogramming in the germ line. Epigenetics. 2008;3: 5–13. 10.4161/epi.3.1.5553 18259118

[135] Reik W, Dean W, Walter J. Epigenetic reprogramming in mammalian development. Science. 2001;293: 1089–1093. 10.1126/science.1063443 11498579

[136] Jacobs MN, Marczylo EL, Guerrero-Bosagna C, Rüegg J. Marked for Life: epigenetic effects of endocrine disrupting chemicals. Annu Rev Environ Resour. 2017;42: 105–160.

[137] Szyf M. Nongenetic inheritance and transgenerational epigenetics. Trends Mol Med. 2015;21: 134–144. 10.1016/j.molmed.2014.12.004 25601643

[138] Hao C, Gely-Pernot A, Kervarrec C, Boudjema M, Becker E, Khil P, et al Exposure to the widely used herbicide atrazine results in deregulation of global tissue-specific RNA transcription in the third generation and is associated with a global decrease of histone trimethylation in mice. Nucleic Acids Res. 2016;44: 9784–9802. 10.1093/nar/gkw840 27655631 PMC5175363

[139] McBirney M, King SE, Pappalardo M, Houser E, Unkefer M, Nilsson E, et al Atrazine induced epigenetic transgenerational inheritance of disease, lean phenotype and sperm epimutation pathology biomarkers. PloS One. 2017;12: e0184306 10.1371/journal.pone.0184306 28931070 PMC5606923

[140] Cleary JA, Tillitt DE, vom Saal FS, Nicks DK, Claunch RA, Bhandari RK. Atrazine induced transgenerational reproductive effects in medaka (*Oryzias latipes*). Environ Pollut. 2019;251: 639–650. 10.1016/j.envpol.2019.05.013 31108297

