# Supplementary material for: Paternal exposure to a common herbicide alters the behavior and serotonergic system of zebrafish offspring
Source: PLoS One. 2020 Apr 10;15(4):e0228357. doi: 10.1371/journal.pone.0228357 (PMC7147785; doi:10.1371/journal.pone.0228357)
Supplement: S1 File — Details of supplementary methods including ethovision setup used for behavioral testing (Fig A in S1 File), primers used (Table A in S1 File), as well as supplementary results including treatment effects on transcript number (Fig B in S1 File) and model outputs (Tables B, C and D in S1 File). (DOCX) [file pone.0228357.s001.docx]

**Paternal exposure to a common herbicide alters the behavior and serotonergic system of zebrafish offspring supplementary material**

Simon D. Lamb^1*¶^, Jolyn H.Z. Chia^1¶^, Sheri L. Johnson^1¶^

^1^Department of Zoology, University of Otago, Dunedin, Otago, New Zealand

^*^Corresponding author:

Simon D. Lamb

Email: [simon.lamb001@gmail.com](mailto:simon.lamb001@gmail.com) (SDL)

For future correspondence:

Sheri L. Johnson

Email: sheri.johnson@otago.ac.nz

Tel: +64 (0) 3 479 7929

**I. Supplemental Methods**


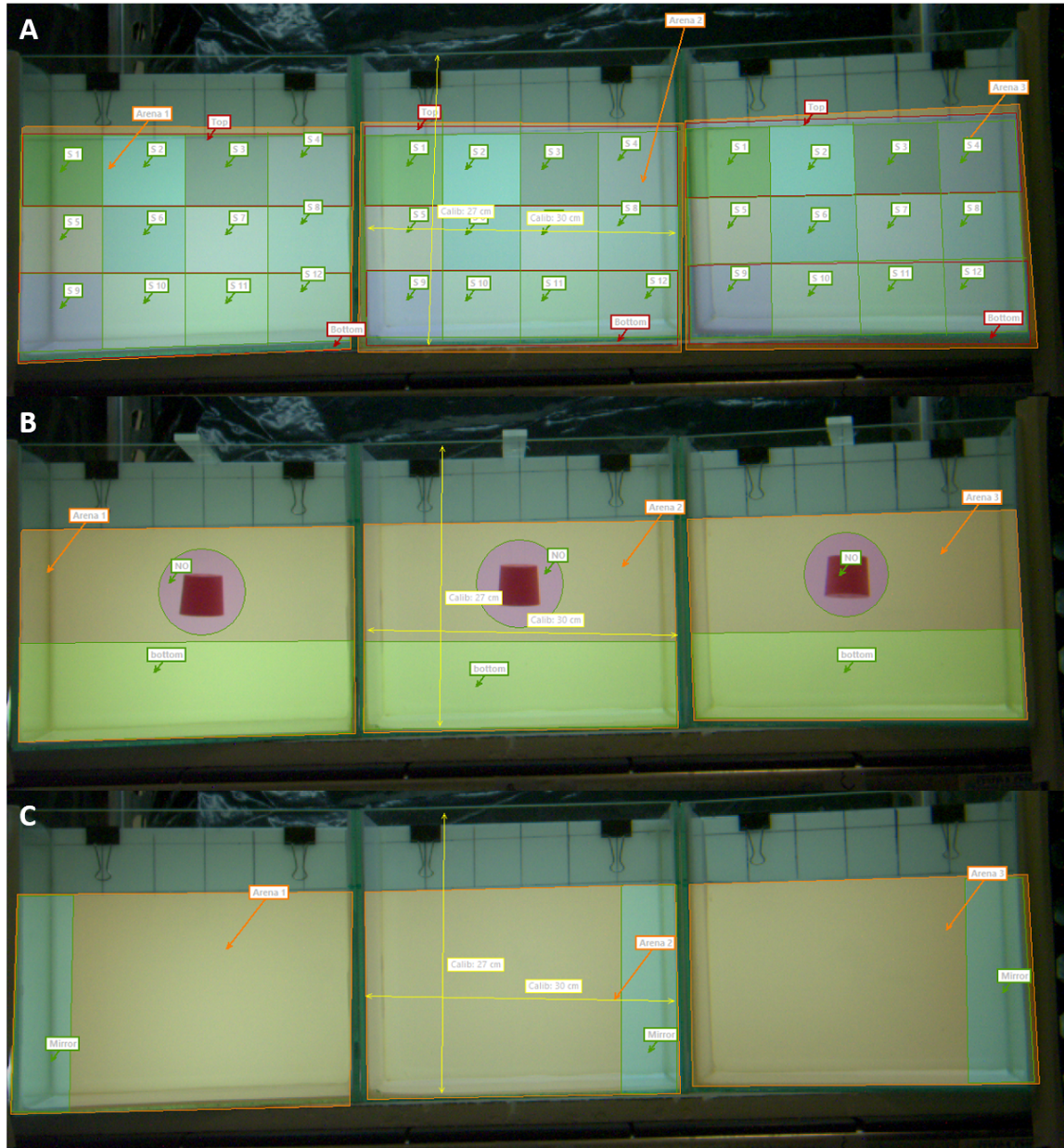


**SA Fig. Ethovision settings.** The settings for quantifying behaviors during the A) novel arena test; B) novel object test; C) mirror test. Within EthoVision XT, for the novel arena test, a four by three square grid is superimposed over the three test tanks. Each differently colored square indicates a different zone (of ∼5 cm wide and long). For the novel object test, a circular purple zone, measuring ∼1.5 body lengths is superimposed over the novel object that hangs from the top of the tank. A green zone superimposed onto the tank indicates the bottom zone. For the mirror test, a blue zone measuring ∼5cm wide, along the side of the tank indicates the mirror zone, where the flat mirror was located.

**SA Table.** **Primers used for qPCR analysis.** Primers used for qPCR analysis, validated by Theodoridi et al., (2017).

| Gene | Direction | Primer Sequence | Annealing temperature |
| --- | --- | --- | --- |
| *b-actin* | Forward primer | 5’ TGTCCCTGTATGCCTCTGGT 3’ | 62 ^o^C |
|  | Reverse primer | 5’ AAGTCCAGACGGAGGATGG 3’ |  |
| *slc6a4a* | Forward primer | 5’ GTCTCCAATGGTTATCGCAGTA 3’ | 60 ^o^C |
|  | Reverse primer | 5’ GATGACCGACAACAGGAAGT 3’ |  |
| *slc6a4b* | Forward primer | 5’ GAATCCTCTGGGCTTGGTAATG 3’ | 60 ^o^C |
|  | Reverse primer | 5’ GCTGAAGTAGACAATGGTGAAGAT 3’ |  |
| *htr1Aa* | Forward primer | 5’ CAGAGCAGAGCAGCACAAG 3’ | 60 ^o^C |
|  | Reverse primer | 5’ TGGTCTGAGAGTTCTGGTCTAATC 3’ |  |
| *htr1B* | Forward primer | 5’ GTGTCGGTGCTCGTGATG 3’ | 60 ^o^C |
|  | Reverse primer | 5’ CAGCCAGATGTCGCAGATG 3’ |  |
| *htr2B* | Forward primer | 5’ GCTGCTCATTCTTCTGGTCAT 3’ | 60 ^o^C |
|  | Reverse primer | 5’ GTTAGTGGCGTTCTGGAGTT 3’ |  |

**II. Supplementary Results**

**SB Table.** **The effects of paternal atrazine exposure (at 0.3ppb, 3ppb or 30ppb) on behavior of unexposed zebrafish offspring model outputs.** The effects of paternal atrazine exposure (at 0.3ppb, 3ppb or 30ppb) on behavior of unexposed zebrafish offspring (time in the bottom zone during the novel arena test (s), time in the bottom zone during the novel object test (s), latency to enter the top zone of the novel arena (s), proportion that approached the novel object, the standard deviation of exploration (s), the time spent interacting with the mirror (sec), the total distance moved (cm) in the novel arena test, novel object test and mirror test). Model estimates, variance (standard errors of fixed effects and variance estimates of random effects), lower and upper 95% confidence intervals, test-statistics (Z or t) and significance levels (P) are from linear and generalized mixed effects models. Significant parameter values are presented in bold.

| Response | Estimate | Variance | Lwr95%CI | Upr95%CI | Z/t | P |
| --- | --- | --- | --- | --- | --- | --- |
| Time in the bottom zone of the novel arena test (sec) | | | | | | |
| Intercept | 274.1 | 65.59 | 145.54 | 402.65 | 4.18 | 0.003 |
| 0.3ppb | 134.84 | 92.05 | −45.57 | 315.25 | 1.47 | 0.181 |
| 3ppb | 65.14 | 91.92 | −115.01 | 245.30 | 0.71 | 0.499 |
| 30ppb | 69.96 | 91.98 | −110.33 | 250.24 | 0.76 | 0.469 |
| Sex(male) | **−86.78** | **21.19** | **−128.30** | **−45.25** | **−4.10** | **<0.001** |
| Random effects |  |  |  |  |  |  |
| Family |  | 11425 |  |  |  |  |
|  |  |  |  |  |  |  |
| Time in the bottom zone of the novel object test (sec) | | | | | | |
| Intercept | 319.97 | 61.36 | 199.71 | 440.23 | 5.22 | <0.001 |
| 0.3ppb | **180.19** | **85.21** | **13.18** | **347.20** | **2.12** | **0.068*** |
| 3ppb | 163.90 | 85.11 | −2.92 | 330.71 | 1.93 | 0.092 |
| 30ppb | 129.86 | 85.04 | −36.82 | 296.54 | 1.53 | 0.167 |
| Sex(male) | **−78.44** | **30.04** | **−137.31** | **−19.57** | **−2.61** | **0.010** |
| Random effects |  |  |  |  |  |  |
| Family |  | 8297 |  |  |  |  |
|  |  |  |  |  |  |  |
| Latency to enter the top zone of the novel arena (sec) | | | | | | |
| Intercept | 40.80 | 1.00 | 40.45 | 41.26 | 769.60 | <0.001 |
| 0.3ppb | **6.16** | **1.76** | **2.01** | **18.73** | **3.20** | **0.001** |
| 3ppb | **4.07** | **1.76** | **1.34** | **12.30** | **2.50** | **0.013** |
| 30ppb | **3.71** | **1.76** | **1.22** | **11.25** | **2.30** | **0.021** |
| Sex(male) | **−2.30** | **1.00** | **−2.32** | **−2.27** | **−172.80** | **<0.001** |
| Random effects |  |  |  |  |  |  |
| Family |  | 1.85 |  |  |  |  |
|  |  |  |  |  |  |  |
| Proportion that approached the novel object | | | | | | |
| Intercept | 0.13 | 0.62 | −0.05 | −0.28 | −3.87 | <0.001 |
| 0.3ppb | 0.63 | 0.65 | −0.34 | 0.85 | 0.87 | 0.386 |
| 3ppb | 0.59 | 0.65 | −0.30 | 0.83 | 0.58 | 0.565 |
| 30ppb | −0.48 | 0.65 | −0.22 | 0.76 | −0.11 | 0.913 |
| Sex(male) | **0.87** | **0.59** | **0.76** | **0.93** | **5.03** | **<0.001** |
| Random effects |  |  |  |  |  |  |
| Family |  | 0.1831 |  |  |  |  |
|  |  |  |  |  |  |  |
| The standard deviation of exploration (sec) | | | | | | |
| Intercept | 59.55 | 4.50 | 50.73 | 68.38 | 13.23 | <0.001 |
| 0.3ppb | 5.80 | 5.93 | −5.82 | 17.42 | 0.98 | 0.329 |
| 3ppb | −1.27 | 5.86 | −12.75 | 10.21 | −0.22 | 0.828 |
| 30ppb | 0.87 | 5.89 | −10.66 | 12.41 | 0.15 | 0.882 |
| Sex(male) | **−12.43** | **4.26** | **−20.77** | **−4.09** | **−2.92** | **0.004** |
| Random effects |  |  |  |  |  |  |
| Family |  | 0.0 |  |  |  |  |
|  |  |  |  |  |  |  |
| The time spent interacting with the mirror (sec) | | | | | | |
| Intercept | 256.68 | 25.08 | 207.53 | 305.83 | 10.24 | <0.001 |
| 0.3ppb | **−110.63** | **33.02** | **−175.35** | **−45.91** | **−3.35** | **<0.001** |
| 3ppb | −56.32 | 32.62 | −120.25 | 7.61 | −1.73 | 0.086 |
| 30ppb | **−95.98** | **32.79** | **−160.24** | **−31.72** | **−2.93** | **0.004** |
| Sex(male) | **74.42** | **23.71** | **27.96** | **120.89** | **3.14** | **0.002** |
| Random effects |  |  |  |  |  |  |
| Family |  | 0.00 |  |  |  |  |
|  |  |  |  |  |  |  |
| The total distance moved in the novel arena (cm) | | | | | | |
| Intercept | 3005.79 | 213.30 | 2587.73 | 3423.85 | 14.09 | <0.001 |
| 0.3ppb | −268.97 | 284.21 | −826.02 | 288.08 | −0.95 | 0.373 |
| 3ppb | −143.93 | 281.27 | −695.22 | 407.35 | −0.51 | 0.624 |
| 30ppb | −481.99 | 282.56 | −1035.79 | 71.82 | −1.71 | 0.129 |
| Sex(male) | **1255.64** | **185.46** | **892.14** | **1619.14** | **6.77** | **<0.001** |
| Random effects |  |  |  |  |  |  |
| Family |  |  |  |  |  |  |
|  |  |  |  |  |  |  |
| The total distance moved in the novel object test (cm) | | | | | | |
| Intercept | 1204.45 | 195.45 | 821.37 | 1587.53 | 6.16 | <0.001 |
| 0.3ppb | 367.21 | 259.14 | −140.71 | 875.12 | 1.42 | 0.194 |
| 3ppb | 177.76 | 258.55 | −328.99 | 684.52 | 0.69 | 0.511 |
| 30ppb | 195.99 | 257.38 | −308.46 | 700.45 | 0.76 | 0.469 |
| Sex(male) | **1257.18** | **173.18** | **917.76** | **1596.60** | **7.26** | **<0.001** |
| Random effects |  |  |  |  |  |  |
| Family |  | 13634 |  |  |  |  |
|  |  |  |  |  |  |  |
| The total distance moved in the mirror test (cm) | | | | | | |
| Intercept | 2205.50 | 159.50 | 1892.89 | 2518.20 | 13.83 | <0.001 |
| 0.3ppb | **−448.20** | **210.10** | **−859.95** | **−36.53** | **−2.13** | **0.034** |
| 3ppb | −368.70 | 207.50 | −775.41 | 37.91 | −1.78 | 0.077 |
| 30ppb | **−500.10** | **208.60** | **−908.81** | **−91.30** | **−2.40** | **0.018** |
| Sex(male) | **689.30** | **150.80** | **393.78** | **984.89** | **4.571** | **<0.001** |
| Random effects |  |  |  |  |  |  |
| Family |  | ∼0 |  |  |  |  |

* = Note, while the p-value is >0.05, the confidence interval does not cross 0, and is thus considered significantly different.

**Additional effects of atrazine on mRNA transcript number**

Paternal atrazine exposure did not significantly alter the mRNA transcript numbers of any of the genes tested compared to controls (Fig. S2; Table S3). Males overall exhibited significantly lower *htr1Aa* mRNA transcripts (Est. –0.47 [–0.94, –0.002 95 % CI]), however this effect was likely driven by the higher transcripts observed in the control females. Indeed, control females exhibited higher *htr1Aa* mRNA transcripts compared to control males, but amongst fish paternally exposed to atrazine this pattern appeared to be reversed between the sexes (Fig. S2) suggesting a marginally non-significant, interactive effect. A similar pattern was observed with *slc6a4a* transcripts, but this too was statistically non-significant (Fig. S2).


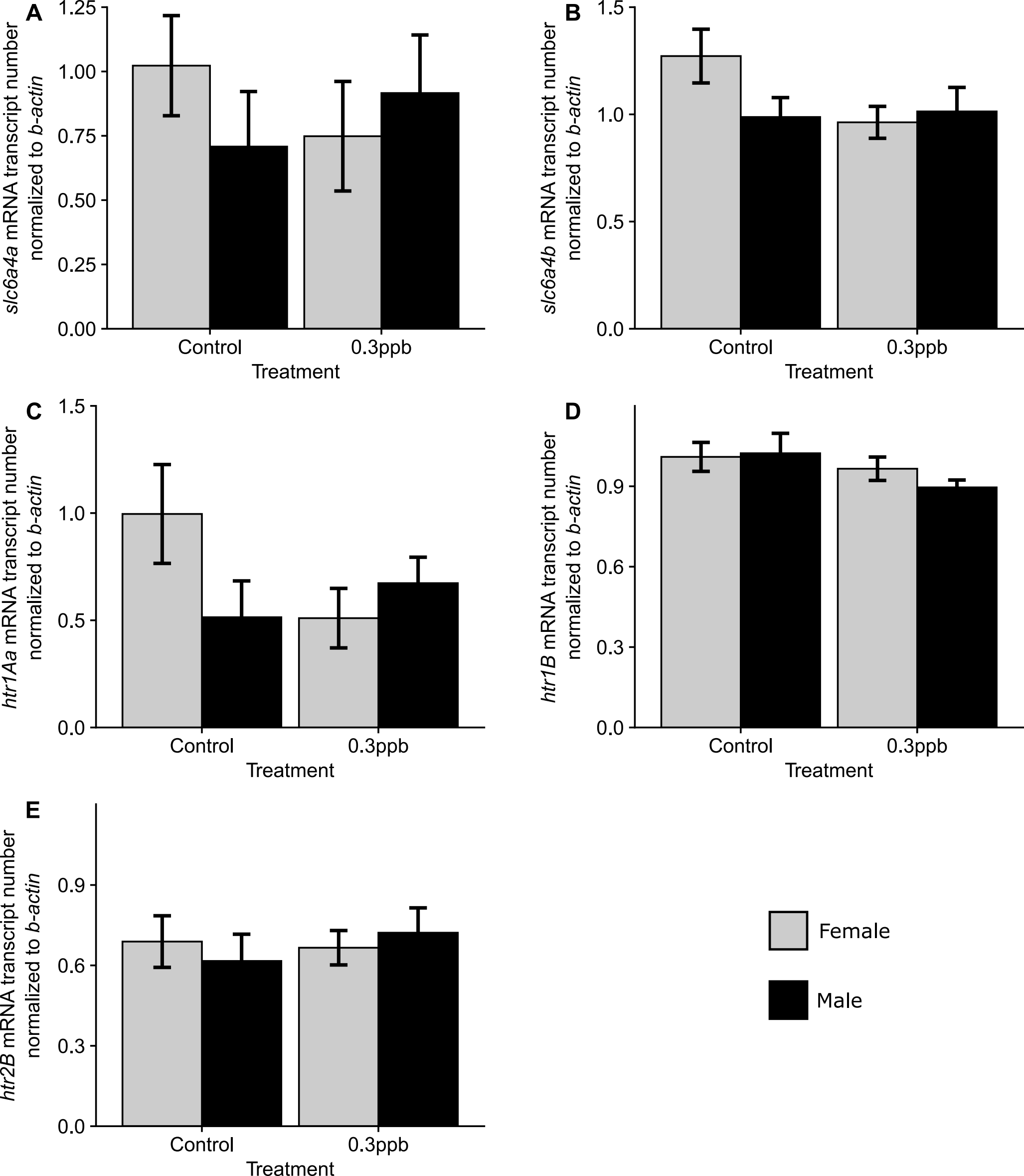


**SB Fig.** **The effects of paternal atrazine exposure (at 0.3ppb) on mRNA transcript number at genes involved in regulating the serotonergic system**. The effects of paternal atrazine exposure (at 0.3ppb) on mRNA transcript number at genes involved in regulating the serotonergic system, (A) *slcl6a4a* (B) *slc6a4b* (C) *htr1Aa* D) *htr1B* and (E) *htr2B*. Bars represent means, with error bars representing standard errors of the mean.

**SC Table. The effects of paternal atrazine exposure (at 0.3ppb) on mRNA transcript number at genes involved in regulating the serotonergic system.** The effects of paternal atrazine exposure (at 0.3ppb) on mRNA transcript number at *slcl6a4a*, *slc6a4b*, *htr1Aa*, *htr1B* and *htr2B*. Model estimates, variance (standard errors of fixed effects and variance estimates of random effects), lower and upper 95% confidence intervals (CI), test-statistics (T) and p-values (P) are from linear mixed effects models. Significant parameter values are presented in bold.

| Response | Estimate | Variance | Lwr95%CI | Upr95%CI | T | P |
| --- | --- | --- | --- | --- | --- | --- |
| *slc6a4a* | | | | | | |
| Intercept | 0.91 | 0.25 | 0.41 | 1.40 | 3.60 | 0.013 |
| Treatment(0.3ppb) | –0.04 | 0.33 | –0.69 | 0.61 | –0.13 | 0.903 |
| Sex(male) | –0.06 | 0.20 | –0.45 | 0.32 | –0.32 | 0.749 |
| Random effects |  |  |  |  |  |  |
| Family |  | 0.105 |  |  |  |  |
|  |  |  |  |  |  |  |
| *slc6a4b* |  |  |  |  |  |  |
| Intercept | 1.19 | 0.09 | 1.02 | 1.37 | 13.20 | <0.001 |
| Treatment(0.3ppb) | –0.15 | 0.11 | –0.36 | 0.06 | –1.41 | –0.165 |
| Sex(male) | –0.19 | 0.11 | –0.32 | 0.10 | –1.03 | 0.310 |
| Random effects |  |  |  |  |  |  |
| Family |  | 0 |  |  |  |  |
|  |  |  |  |  |  |  |
| *htr1Aa* |  |  |  |  |  |  |
| Intercept | 1.00 | 0.19 | 0.62 | 1.38 | 5.15 | <0.001 |
| Treatment(0.3ppb) | –0.49 | 0.27 | –1.02 | 0.05 | 1.78 | 0.110 |
| Sex(male) | **–0.47** | **0.24** | **–0.94** | **–0.002** | **–1.97** | **0.056*** |
| Treatment(0.3ppb):Sex(male) | 0.63 | 0.33 | –0.01 | 1.28 | 1.92 | 0.062 |
| Random effects |  |  |  |  |  |  |
| Family |  | 0.035 |  |  |  |  |
|  |  |  |  |  |  |  |
| *htr1B* |  |  |  |  |  |  |
| Intercept | 1.03 | 0.09 | 0.86 | 1.19 | 12.02 | <0.001 |
| Treatment(0.3ppb) | –0.08 | 0.12 | –0.31 | 0.16 | –0.64 | 0.558 |
| Sex(male) | –0.04 | 0.04 | –0.11 | 0.03 | –1.04 | 0.307 |
| Random effects |  |  |  |  |  |  |
| Family |  | 0.019 |  |  |  |  |
|  |  |  |  |  |  |  |
| *htr2B* |  |  |  |  |  |  |
| Intercept | 0.70 | 0.10 | 0.46 | 0.86 | 6.43 | <0.001 |
| Treatment(0.3ppb) | 0.04 | 0.13 | -0.23 | 0.30 | 0.27 | 0.800 |
| Sex(male) | –0.003 | 0.08 | -0.16 | 0.16 | –0.04 | 0.970 |
| Random effects |  |  |  |  |  |  |
| Family | 0.017 |  |  |  |  |  |

* = Note, while the p-value is >0.05, the confidence interval does not cross 0, and is thus considered significantly different.

**SD Table. The effects of paternal atrazine exposure (at 0.3ppb) on the relationship between time spent in the bottom zone of the novel arena (s) and mRNA transcript number for genes involved in regulating the serotonergic system.** The effects of paternal atrazine exposure (at 0.3ppb) on the relationship between time spent in the bottom zone of the novel arena (s) and mRNA transcript number for genes involved in regulating the serotonergic system (*slcl6a4a*, *slc6a4b, htr1Aa*, *htr1B*, *htr2B*). Model estimates, variance (standard errors of fixed effects and variance estimates of random effects), lower and upper 95% confidence intervals (CI), test-statistics (T) and p-values (P) are from linear mixed effects models. Significant parameter values are presented in bold.

| Response | Estimate | Variance | Lwr95%CI | Upr95%CI | T | P |
| --- | --- | --- | --- | --- | --- | --- |
| Time spent in the bottom zone of the novel arena (sec) | | | | | | |
| Intercept | 245.27 | 42.00 | 162.95 | 327.59 | 5.84 | <0.001 |
| c.slc6a4a | 129.43 | 63.34 | 5.28 | 253.58 | 2.04 | 0.047 |
| Treatment(0.3ppb) | 119.03 | 58.14 | 5.08 | 232.99 | 2.05 | 0.047 |
| c.log *slc6a4a*:  Treatment(0.3ppb) | **−191.46** | **83.79** | **−355.69** | **−27.23** | **−2.29** | **0.027** |
| Random effects |  |  |  |  |  |  |
| Family |  | 0 |  |  |  |  |
|  |  |  |  |  |  |  |
| Intercept | 243.75 | 44.41 | 156.71 | 330.78 | 5.49 | <0.001 |
| c. *slc6a4b* | 58.64 | 86.48 | −110.85 | 228.14 | 0.68 | 0.501 |
| Treatment(0.3ppb) | 126.29 | 62.17 | 4.44 | 248.14 | 2.03 | 0.048 |
| Random effects |  |  |  |  |  |  |
| Family |  | 0 |  |  |  |  |
|  |  |  |  |  |  |  |
| Intercept | 232.24 | 40.58 | 152.69 | 311.78 | 5.72 | <0.001 |
| c. *htr1Aa* | 167.80 | 57.22 | 55.66 | 279.94 | 2.93 | 0.005 |
| Treatment(0.3ppb) | 125.26 | 56.47 | 14.58 | 235.94 | 2.22 | 0.032 |
| c.log *htr1Aa*:  Treatment(0.3ppb) | **−260.37** | **104.60** | **−465.39** | **−55.35** | **−2.49** | **0.017** |
| Random effects |  |  |  |  |  |  |
| Family |  | 0 |  |  |  |  |
|  |  |  |  |  |  |  |
| Intercept | 253.45 | 46.66 | 161.99 | 344.91 | 5.43 | 0.009 |
| c. *htr1B* | −102.69 | 187.08 | −469.36 | 263.97 | −0.55 | 0.593 |
| Treatment(0.3ppb) | 108.07 | 65.64 | −20.57 | 236.72 | 1.65 | 0.191 |
| Random effects |  |  |  |  |  |  |
| Family |  | 572.1 |  |  |  |  |
|  |  |  |  |  |  |  |
| Intercept | 247.91 | 44.10 | 161.46 | 334.3495 | 5.62 | <0.001 |
| c. *htr2B* | −28.37 | 105.33 | −234.80 | 178.07 | −0.27 | 0.789 |
| Treatment(0.3ppb) | 118.32 | 61.12 | −1.48 | 238.11 | 1.94 | 0.060 |
| Random effects |  |  |  |  |  |  |
| Family |  | 0 |  |  |  |  |

**III. Supplemental References**

Noldus, L.P., Spink, A.J., and Tegelenbosch, R.A. (2001). EthoVision: a versatile video tracking system for automation of behavioral experiments. Behav. Res. Methods Instrum. Comput. *33*, 398–414.

Theodoridi, A., Tsalafouta, A., and Pavlidis, M. (2017). Acute exposure to fluoxetine alters aggressive behavior of zebrafish and expression of genes involved in serotonergic system regulation. Front. Neurosci. *11*, 223.
